# Walking control of humanoid robots based on improved footstep planner and whole-body coordination controller

**DOI:** 10.3389/fnbot.2025.1538979

**Published:** 2025-02-21

**Authors:** Xiangji Wang, Wei Guo, Siyu Yin, Sen Zhang, Fusheng Zha, Mantian Li, Pengfei Wang, Xiaolin Li, Lining Sun

**Affiliations:** ^1^State Key Laboratory of Robotics and System Harbin Institute of Technology, Harbin, China; ^2^Lanzhou University of Technology, Lanzhou, China; ^3^Institute of Intelligent Manufacturing Technology, Shenzhen Polytechnic University, Shenzhen, China

**Keywords:** humanoid robot, footstep planner, linear inverted pendulum, model predictive control, whole-body coordination control

## Abstract

High-speed walking is fundamental for humanoid robots to quickly reach the work site in emergency scenarios. According to biological studies, the coordinated motion of the arms and waist can significantly enhance walking speed and stability in humans. However, existing humanoid robot walking control frameworks predominantly focus on leg control, often overlooking the utilization of upper body joints. In this paper, a novel walking control framework combining the improved footstep planner and the whole-body coordination controller is proposed, aiming to improve the humanoid robot's tracking accuracy of desired speeds and its dynamic walking capability. First, we analyze the issues in traditional footstep planners based on Linear Inverted Pendulum and Model Predictive Control (LIP-MPC). By reconstructing the footstep optimization problem during walking using the Center-of-Mass (CoM) position, we propose an improved footstep planner to enhance the control accuracy of the desired walking speed in humanoid robots. Next, based on biological research, we define a coordinated control strategy for the arms and waist during walking. Specifically, the waist increases the robot's step length, while the arms counteract disturbance momentum and maintain balance. Based on the aforementioned strategy, we design a whole-body coordination controller for the humanoid robot. This controller adopts a novel hierarchical design approach, in which the dynamics and motion controllers for the upper and lower body are modeled and managed separately. This helps avoid the issue of poor control performance caused by multi-task coupling in traditional whole-body controllers. Finally, we integrate these controllers into a novel walking control framework and validate it on the simulation prototype of the humanoid robot Dexbot. Simulation results show that the proposed framework significantly enhances the maximum walking capability of the humanoid robot, demonstrating its feasibility and effectiveness.

## 1 Introduction

Walking serves as the foundation for humanoid robots to operate stably in various environments, its dynamics and robustness directly affect the robot's work efficiency. With the significant advancement of humanoid robot structures and actuators, many researchers have conducted in-depth studies on leg control and stable walking control methods. Although numerous impressive results have been achieved, these methods still struggle to support high-speed walking for humanoid robots in emergency scenarios (Xiaobin and Aaron, 2022; Yu-Ming et al., 2024). Therefore, further enhancing the dynamic performance and disturbance rejection capability of humanoid walking has become a key objective in humanoid robot research. According to bionic studies, humans primarily counteract self-induced or external disturbances during walking by adjusting footstep placement and coordinating whole-body limb movements (Horak and Nashner, 1986; Thierry et al., 2001). Inspired by humans, enhancing the dynamic walking capability of humanoid robots requires motion control systems that not only incorporate highly effective footstep planning to regulate the CoM velocity but also enable coordinated control of all joints to maintain stability. Consequently, the development of efficient footstep planning methods and the incorporation of whole-body coordination strategies involving the waist and arms have become key priorities for future humanoid robot research.

The Linear Inverted Pendulum (LIP) and the Spring-Loaded Inverted Pendulum (SLIP) are the most effective simplified models derived from the characteristics of CoM motion in biological movements (George et al., 2023; Xiaolong et al., 2024). The SLIP model, due to its dynamic properties, is commonly applied in the dynamic motion control of quadruped robots, including diagonal trotting, jumping, and galloping gaits (Dominic et al., 2018; Sovukluk et al., 2024). However, its dynamic characteristics pose challenges for humanoid robots with distributed mass, particularly in state estimation and joint impedance control, making it difficult to apply in real-world systems (Hong et al., 2022; Stéphane et al., 2020). The LIP model is widely utilized in the walking planning and control of humanoid robots across complex terrains, such as flat surfaces, slopes, and stairs. A pioneering study combined MPC with the Zero Moment Point (ZMP) approach, using the cart-table model to plan the robot's CoM trajectory, and designed an online controller to track the desired ZMP for stable walking of the humanoid robot (Kajita et al., 2003). However, these methods require high-precision foot sensors to accurately sense contact forces, which are impractical for real-world environments with complex terrains. Additionally, the ZMP stability criterion is not applicable to humanoid robot systems equipped with point or line-shaped feet, which limits its applicability. The MPC algorithm, due to its feedback correction and rolling optimization features, has been widely applied in footstep trajectory generation tasks. Nicola et al. (2020) proposed the Inherently Stable Model Predictive Control (IS-MPC) framework, which uses the LIP dynamics model as the prediction model and ZMP velocity as the control input. This method generates a gait, including time frames, in real-time to achieve omnidirectional motion commands. Elham et al. (2021) implemented stable walking for the small bipedal robot Blot using a hierarchical MPC framework. Guiyang et al. (2021) combined MPC and the LIP dynamics model to create an efficiently computed footstep prediction planner for quick response to disturbances during walking. The MPC controller has become a powerful tool for humanoid robot walking control (Patrick et al., 2023). However, in current applications, there are often issues with large CoM velocity tracking errors and limited iterations, making it difficult to meet the accuracy requirements for tracking desired average speed during humanoid robot walking.

After the robot obtains the optimal footstep positions, the desired CoM trajectory and swing leg trajectory can be obtained through integration of the LIP dynamics equations and polynomial fitting. The primary task of the motion control system is to input optimal joint drive commands to effectively track the specified task trajectory. When a humanoid robot is performing high-speed dynamic walking, the control system faces two main challenges. The first challenge is that, during walking, the robot's step length increases with the desired speed. If the step length is too long, it may cause the robot to reach mechanical limits or result in a smaller adjustable range for foot placement. The swing leg may fail to reach the desired footstep position, preventing the robot from effectively controlling the CoM motion, which could lead to a fall. To address this issue, related biological studies have found through human gait motion capture data that the waist rotates with the swinging of the legs during walking, and its range of rotation increases with the step length. Some robotic researchers have innovatively incorporated this characteristic into motion control systems. Beomyeong et al. (2020) analyzed the impact of the waist on the kinematics of humanoid robots and concluded that the introduction of the waist can enhance the range of motion of the robot's legs. Based on this conclusion, they designed an optimization problem to generate the waist motion trajectory, thereby improving the walking capability of the robot Dyros-Jet. Jehwi et al. (2021) compared the energy consumption and joint torque between fixed waist and coordinated waist motion walking strategies. The results indicated that the coordinated waist walking method could reduce the robot's energy consumption during movement, particularly alleviating the load torque on the knee joints. Hyobin and Inho (2022) introduced the waist into the kinematics of humanoid robot standing tasks, significantly enhancing its flexibility and operational range. Based on the findings from the above studies, it is evident that the effective control of waist motion can improve the range of motion of the robot's legs while reducing energy consumption during walking. The second challenge arises from the fact that as the robot's walking speed increases, the legs need to perform high-frequency, large-amplitude swings to track the desired footstep positions. The reaction forces and torques generated during the swinging motion can cause significant disturbances to the robot's body posture, affecting the stability of the walking process. To address this challenge, some studies have proposed using the waist to drive body rotation in order to compensate for the momentum during walking. However, using torso rotation to balance the disturbance angular momentum leads to large-scale body rotations, which increases the non-linearity of the robot's dynamics. This not only complicates the control but also affects the role the waist must play during the swing leg phase. Since the arms have no external contact forces or specified trajectory tasks during walking, they can swing freely over a wide range. Therefore, using the arms to counteract disturbance momentum is an effective solution. Zhifa et al. (2023) planned the arm swing trajectory, using the arm swing to counteract the disturbances caused by the swinging leg, thereby improving the stability of the bipedal robot during dynamic walking. Dragomir and Ryo (2022) focused on the issue of robotic angular momentum and inherent redundant degrees of freedom, using the angular momentum generated by the swinging arms to counteract external disturbances, thereby enhancing the robot's ability to resist external perturbations. Beomyeong et al. (2020) used the swinging of arms to compensate for the robot's yaw angular momentum during walking, achieving stable walking for the robot. Georg et al. (2016) treated the arms of the Atlas robot as a flywheel system to control the robot's Centroidal Moment Pivot (CMP) and enable real-time tracking of the Instantaneous Capture Point (ICP), achieving walking on a balance beam, a task with extremely high stability requirements. Based on the above research, it is clear that the advantage of using arm swing to counteract disturbances lies in the fact that, during normal walking, the robot's arms do not need to engage in force interaction tasks with the external environment. Moreover, the arm movements are decoupled from the leg movements, allowing for flexible arm swings based on the needs. Unfortunately, the aforementioned studies mainly focus on scenarios such as slow walking or standing in humanoid robots, and do not delve into the arm swing control methods in the context of dynamic walking.

**Figure 1:**
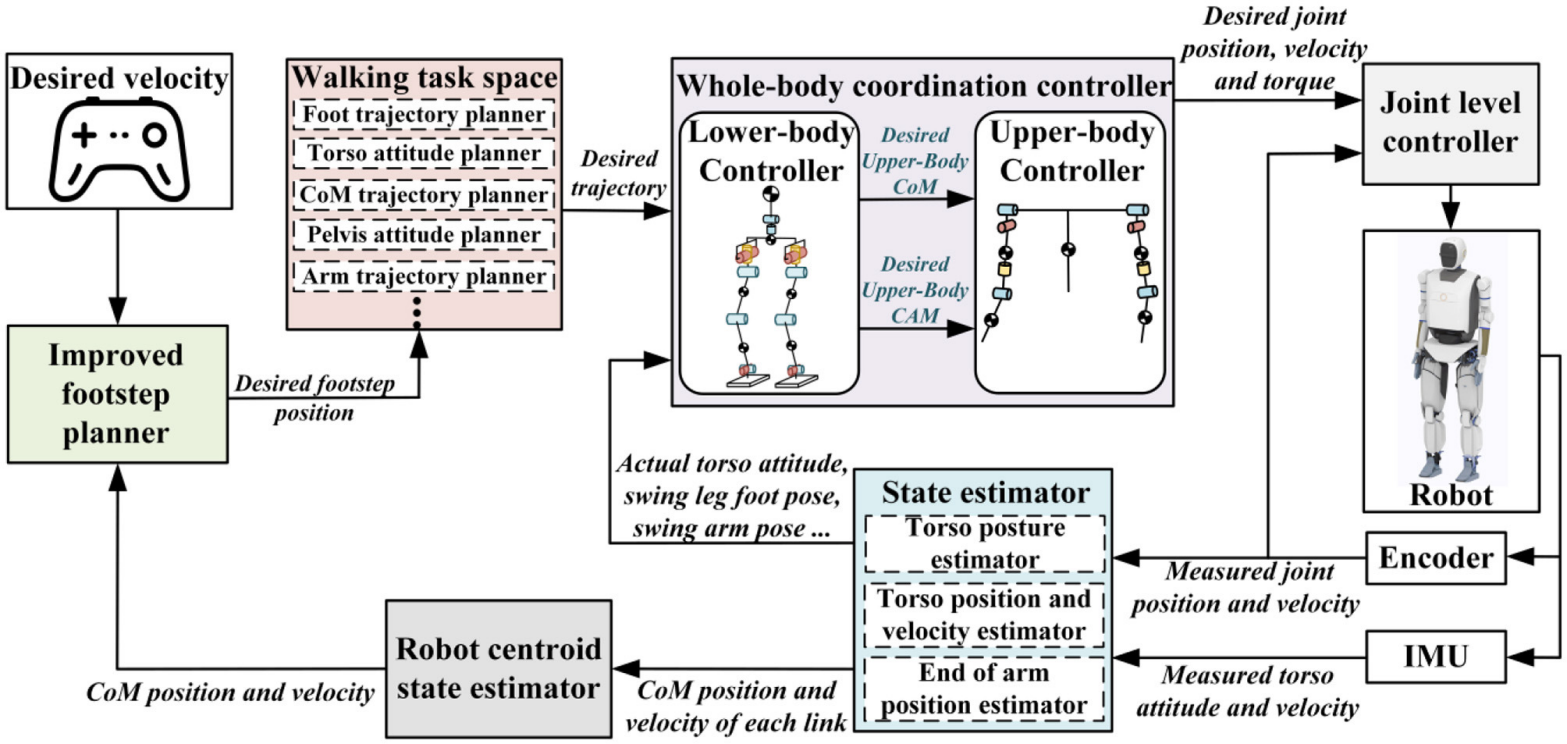
Humanoid robot walking control framework.

Based on the above analysis, this paper aims to enhance the walking capability of humanoid robots by integrating relevant research and conclusions from human gait studies. A walking control framework consisting of the improved footstep planner and the whole-body coordination controller is designed, as shown in Figure 1. In this framework, the improved footstep planner constructs the MPC optimization problem using the position component of the solution to the differential equation in the LIP model. By leveraging the advantages of MPC's rolling optimization, the optimal footstep position can be quickly determined based on the robot's desired walking speed and current motion state. The motion trajectory planner in the walking task space is capable of planning the robot's CoM trajectory, swing leg trajectory, and other whole-body movements based on the desired footstep positions, and then sending these plans to the motion controller for execution. The whole-body coordination controller, based on the given task requirements, controls the robot to achieve stable walking. Compared to existing studies, the walking control framework proposed in this paper deeply draws from human walking strategies, fully utilizing all the degrees of freedom of the humanoid robot, significantly enhancing its dynamic walking capability. The main contributions of this paper are as follows:

1) This paper proposes an improved footstep planner, which is based on an enhancement of the traditional LIP-MPC approach. First, the desired CoM displacement is described using the desired speed and footstep time. Then, the LIP position differential equation solution is used to replace the traditional instantaneous velocity differential for predicting the CoM state. Finally, a quadratic optimization problem is formulated to solve for the robot's optimal footstep position. The improved planner avoids the average speed tracking errors caused by the instantaneous velocity iteration optimization in traditional methods, thereby enhancing the robot's control accuracy over the average walking speed.
2) For dynamic walking of the robot, a coordination strategy for the robot's arms and waist is planned, based on the characteristics of human walking. The whole-body coordination controller is then designed according to the established strategy. The controller adopts a hierarchical WBC design approach, consisting of two components: the Lower-body WBC and the Upper-body WBC. The Lower-body WBC coordinates the robot's legs and waist, fully utilizing the structural advantages of the robot to achieve large-step walking. The Upper-body WBC controller manages the robot's arms to compensate for the centroidal angular momentum. This controller addresses the issue of poor task control performance caused by the coupling of multiple control tasks in humanoid robots with redundant degrees of freedom, significantly enhancing the robot's dynamic walking capability.
3) The improved footstep planner and the whole-body coordination controller are integrated into a humanoid robot walking control framework, and the proposed framework is validated through simulation experiments. The simulation results show that the walking control framework proposed in this paper significantly enhances the robot's dynamic walking ability, demonstrating its feasibility and effectiveness.

The rest of this paper is organized as follows: In Section 2, the derivation of the LIP dynamics model is presented, and the design method for the improved footstep planner is proposed. Section 3 describes the walking control strategy proposed in this paper, and based on this, the design of the whole-body coordination controller is presented. In Section 4, we integrate the improved footstep planner and the whole-body coordination controller, and perform simulation validation. The results demonstrate the advantages of the proposed control framework. Finally, in Section 5, the paper is summarized, and future work is discussed.

## 2 Improved footstep planner

### 2.1 Motion analysis of the LIP model

The LIP model has been widely applied to humanoid robot walking control and has demonstrated excellent control performance. By treating the CoM of a humanoid robot as the CoM of the LIP model, the complex motion of a humanoid robot can be simplified to the simple LIP model (Lim et al., 2012, 2004). Assume that after each footstep, the center of the supporting foot is taken as the current origin of the coordinate system. The intersection of the sagittal with the ground forms the x-axis of the coordinate system, and the intersection of the coronal with the ground forms the y-axis. In the motion process, it is assumed that the CoM of the LIP model has no deviation in the z-axis direction from the desired height and is subjected to a constant thrust of *mg*. Taking the x-axis as an example, in the coordinate system of the contact foot, the dynamics equations can be expressed as:


(1) $$\begin{array}{l} {ẍ}_{\text{com}}=\frac{g}{z_{\text{com}}-p_{\text{z}}}\left(x_{\text{com}}-p_{\text{x}}\right) \end{array}$$

Where *g* is the gravitational acceleration, *z*_*com*_ is the constant height of the robot's CoM, and *p*_*x*_ represents the x-axis footstep coordinates in the current coordinate system. The LIP dynamics model can be treated as a differential equation, and its solution can be obtained as:


(2) $$\begin{array}{l} x\left(t\right)=\text{A}\left(t\right)X_{0}+\text{B}\left(t\right)p_{x} \end{array}$$

Where $X={\left[\begin{array}{cc} x_{com} & {ẋ}_{com} \end{array}\right]}^{T}$ represents the CoM state vector, and *X*_0_ denotes the initial state of the CoM. The coefficients *A*(*t*) and *B*(*t*) are defined as:


(3) $$\begin{array}{l} A\left(t\right)=\left[\begin{array}{cc} \cosh\left(\omega t\right) & \omega^{-1}\sinh\left(\omega t\right)\\ \omega \sinh\left(\omega t\right) & \cosh\left(\omega t\right) \end{array}\right] \end{array}$$

(4) $$\begin{array}{l} B\left(t\right)=\left[\begin{array}{c} 1-\cosh\left(\omega t\right)\\ -\omega \sinh\left(\omega t\right) \end{array}\right] \end{array}$$

(5) $$\begin{array}{l} \omega =\sqrt{\frac{g}{z_{\text{com}}-p_{\text{z}}}} \end{array}$$

From the above equations, the future CoM motion state of the robot satisfying the LIP dynamics model at a specific time can be quickly calculated based on the current CoM state *X*_0_, without requiring complex iterative calculations of the dynamic equations.

**Figure 2:**
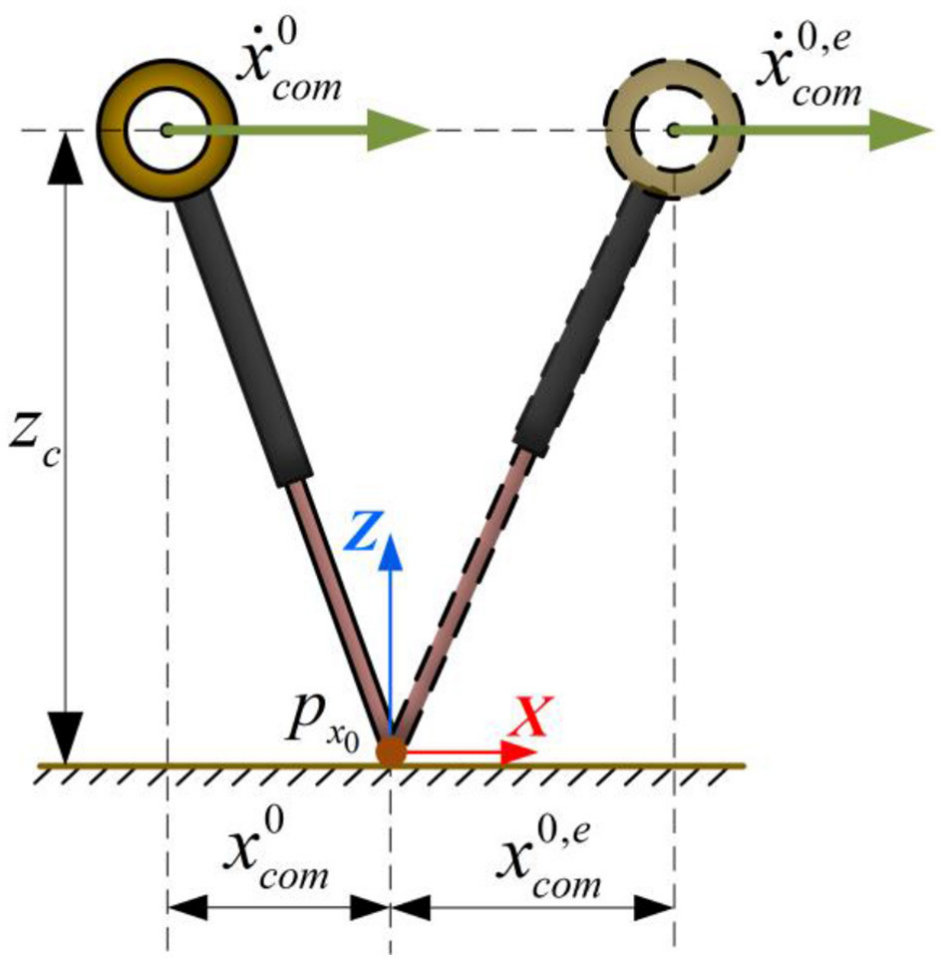
The LIP single-cycle motion process.

Based on the solution of the above LIP dynamics differential equations, the robot's motion state at future time can be quickly computed. The illustration of the LIP single-cycle motion is shown in Figure 2. Given the current contact point coordinates as $p_{x_{0}}$, which is set as the origin of the world coordinate system for the current walking cycle. At the current time *t*_0_, the current CoM position and velocity are $x_{com}^{0}$ and ${ẋ}_{com}^{0}$, respectively. The position and velocity of the CoM at the end of the support phase at time $t_{e}^{0}$ can be expressed as $x_{com}^{0,e}$ and ${ẋ}_{com}^{0,e}$, respectively. The equations can be written as:


(6) $$\begin{array}{l} X\left(t_{e}^{0}\right)=\left[\begin{array}{c} x_{com}^{0,e}\\ {ẋ}_{com}^{0,e} \end{array}\right]=A\left(t_{e}^{0}-t_{0}\right)\left[\begin{array}{c} x_{com}^{0}\\ {ẋ}_{com}^{0} \end{array}\right]+B\left(t_{e}^{0}-t_{0}\right)p_{x_{0}} \end{array}$$

When a humanoid robot performs dynamic walking, the double-support phase is typically neglected. The left and right feet alternately execute a single-support phase according to the set motion cycle, generating reaction forces to maintain the robot's walking. Assuming no significant collision or energy loss during the support foot transition, the CoM motion state at the end of the current support phase and the beginning of the next support phase can be considered equal, which can be expressed as:


(7) $$\begin{array}{l} X_{n}^{-}=X_{n+1}^{+} \end{array}$$

Where n denotes the future n-th step, the superscript “–” indicates the end time of the current support phase, and the superscript “+” indicates the start time of the next support phase. Equation 7 can be further expressed as:


(8) $$\begin{array}{l} \left[\begin{array}{c} x_{com}^{1^{+}}\\ {ẋ}_{com}^{1^{+}} \end{array}\right]=X_{1}^{+}=X_{0}^{-}=A\left(t_{e}^{0}-t_{0}\right)X_{0}+B\left(t_{e}^{0}-t_{0}\right)p_{x_{0}} \end{array}$$

This allows us to obtain the CoM state at the end of the current support phase and at the beginning of the next step's support phase. Expanding the above equation, the motion state of the robot at the n-th future step in the current coordinate system can be expressed as:


(9) $$\begin{array}{l} \;{X_{2}}^{+}={X_{1}}^{-}=A\left(T_{\text{s}}^{\text{1}}\right){X_{1}}^{+}+B\left(T_{\text{s}}^{\text{1}}\right)p_{{\text{x}}_{\text{1}}}^{*}\\ \;{X_{3}}^{+}={X_{2}}^{-}=A\left(T_{\text{s}}^{\text{2}}\right){X_{2}}^{+}+B\left(T_{\text{s}}^{\text{2}}\right)p_{{\text{x}}_{\text{2}}}^{*}\\ \;\vdots \\ {X_{n}}^{+}={X_{n}}^{-}=A\left(T_{\text{s}}^{n-\text{1}}\right){X_{n-1}}^{+}+B\left(T_{\text{s}}^{n-\text{1}}\right)p_{{\text{x}}_{n-1}}^{*} \end{array}$$

where $T_{\text{s}}^{n}$ is the duration of the n-th future support phase, also referred to as the footstep time.

### 2.2 MPC framework for footstep adjustment control

In existing research, the traditional LIP-MPC method typically constructs an optimization problem by using the velocity of the CoM state at the end time of each supporting phase as the prediction variable, thereby determining the optimal foot placement. However, based on the analysis of the LIP-based walking process, the CoM velocity exhibits a deceleration followed by acceleration, with varying velocities at different moments during the motion. If the velocity at a certain moment during the walking process is used as the prediction variable, it is difficult to represent the tracking performance of the reference average velocity throughout the entire process. This issue is particularly pronounced when the step frequency is low, as the LIP model experiences significant velocity fluctuations during the motion, resulting in more noticeable tracking errors.

**Figure 3:**
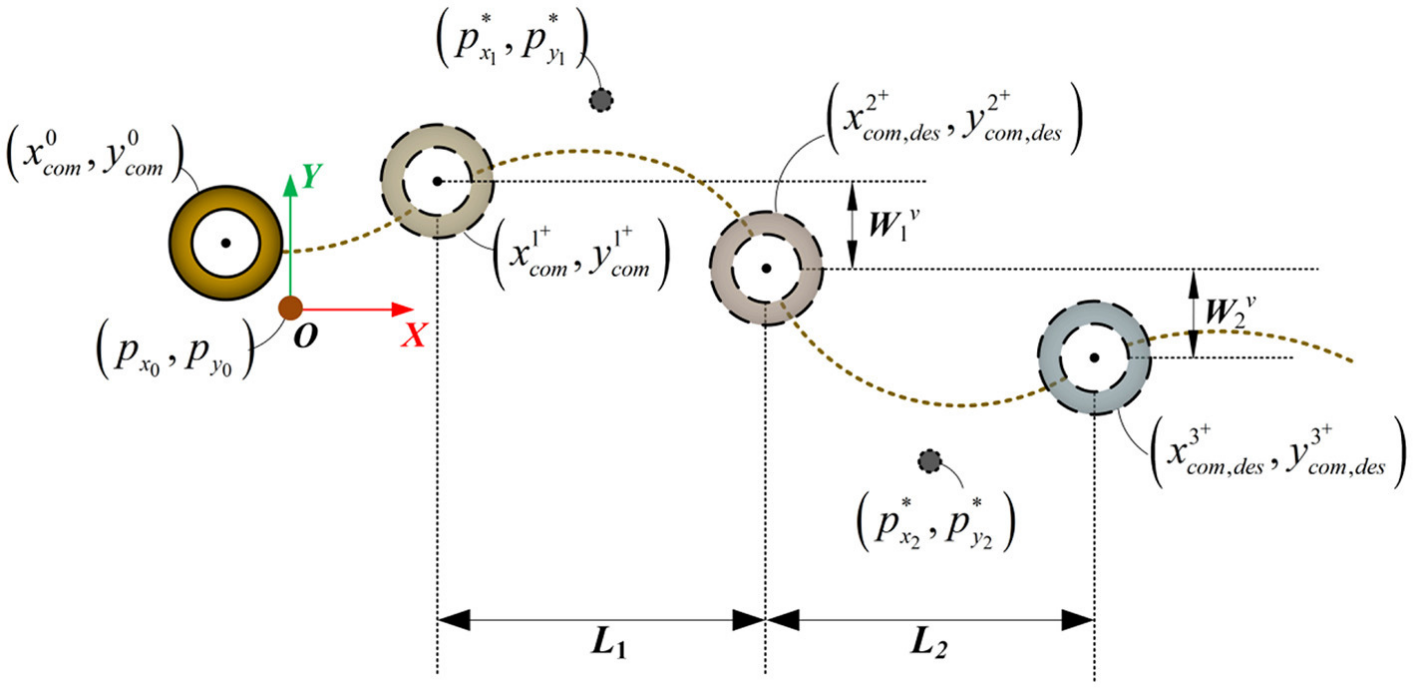
The footstep placement and CoM trajectory in the prediction process of the LIP model.

To avoid the impact of instantaneous velocity on the tracking performance, we abandon the traditional optimization method that uses the CoM velocity at the end of the support phase for tracking. Instead, we utilize the CoM position at the end of the support phase as the desired outcome for the optimization problem to improve the robot's foot placement. The advantage of this strategy lies in the fact that, firstly, it maps the LIP motion velocity to the CoM position and footstep time, enabling more accurate tracking of the desired velocity. Additionally, the CoM position provides a more accurate and reliable state estimation compared to the velocity. The predicted trajectory of the CoM motion and the foot placement are illustrated in Figure 3. Based on this illustration, we will describe the details from both the x-z and the y-z.

#### 2.2.1 x-z plane

In the x-z plane, the LIP model tracks the robot's desired walking speed along the x-axis using the foot placement. When the robot's desired walking speed is set to ẋ_*des*_, and the single-leg support phase for the future n-th step is $T_{\text{s}}^{n}$, the step length for the future n-th step can be estimated as:


(10) $$\begin{array}{l} L_{n}={ẋ}_{des}T_{\text{s}}^{n} \end{array}$$

The x-axis position of the CoM at the end of the support phase during the n-th step can be expressed as:


(11) $$\begin{array}{l} x_{com,des}^{n^{-}}=x_{com,des}^{{\left(n+1\right)}^{+}}=x_{com,des}^{n^{+}}+L_{n} \end{array}$$

It is assumed that after the robot places its foot, there is no relative displacement between the foot and the ground, and no adjustment can be made. Therefore, during the current support phase, the desired CoM state at the end of the phase is the same as the actual CoM state.


(12) $$\begin{array}{l} \left[\begin{array}{c} x_{com}^{0^{-}}\\ {ẋ}_{com}^{0^{-}} \end{array}\right]=X_{0}^{-}=A\left(t_{e}^{0}-t_{0}\right)X_{0}+B\left(t_{e}^{0}-t_{0}\right)p_{x_{0}} \end{array}$$

(13) $$\begin{array}{l} x_{com,des}^{1^{+}}=x_{com}^{0^{-}} \end{array}$$

**Figure 4:**
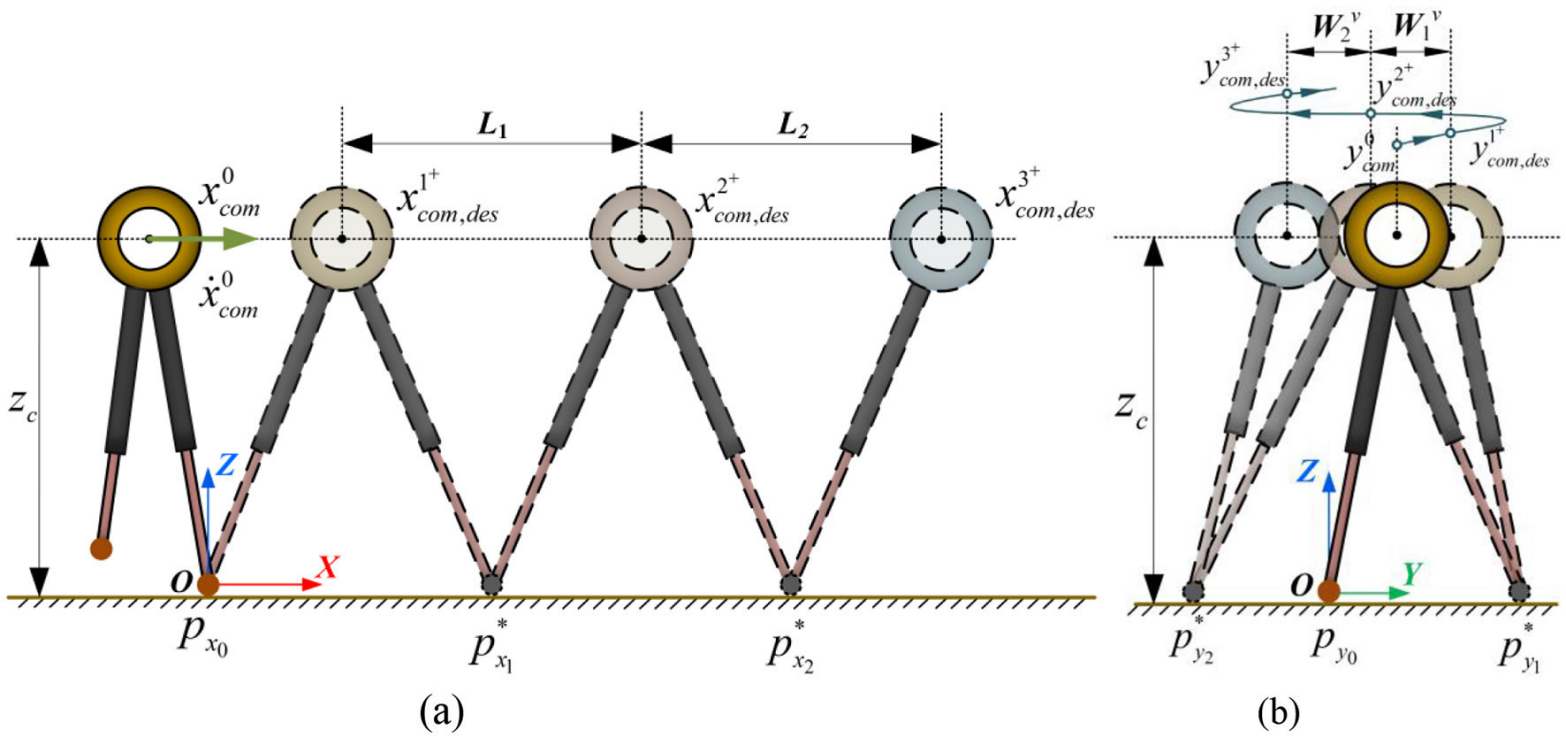
The footstep placement and CoM trajectory in the prediction process of the LIP model in x-z plane and y-z plane. **(A)** x-z plane. **(B)** y-z plane.

Thus, based on the desired velocity, the expected position at the end of each support phase can be iteratively calculated. The illustration of the expected position along the x-axis is shown in Figure 4A.


(14) $$\begin{array}{l} \begin{array}{c} x_{com,des}^{2^{+}}=x_{com,des}^{1^{-}}=x_{com,des}^{1^{+}}+{ẋ}_{des}T_{s}^{1}\\ x_{com,des}^{3^{+}}=x_{com,des}^{2^{-}}=x_{com,des}^{2^{+}}+{ẋ}_{des}T_{s}^{2}\\ \vdots \\ x_{com,des}^{n^{+}}=x_{com,des}^{{\left(n-1\right)}^{-}}=x_{com,des}^{{\left(n-1\right)}^{+}}+{ẋ}_{des}T_{s}^{n-1} \end{array} \end{array}$$

Therefore, the cost function for the optimization problem in the x-z plane can be constructed as:


(15) $$\min_{P_{x}^{*}}\sum_{i=1}^{n}\left({\left\|x_{com,des}^{i^{+}}-x_{com}^{i^{+}}\right\|}_{Q_{x}}^{2}+{\left\|p_{x_{i}}^{*}-p_{x_{i}-1}^{*}\right\|}_{R_{x}}^{2}\right)$$

(15-1) $$\begin{array}{l} s\text{.}t\text{. }X_{1}^{+}=A\left(t_{e}^{0}-t_{0}\right)X_{0}+B\left(t_{e}^{0}-t_{0}\right)p_{x_{0}} \end{array}$$

(15-2) $$\begin{array}{l} {X_{i}}^{+}=A\left(T_{\text{s}}^{i-1}\right){X_{i-1}}^{+}+B\left(T_{\text{s}}^{i-1}\right)p_{{\text{x}}_{i-1}}^{*} \end{array}$$

(15-3) $$\begin{array}{l} p_{{\text{x}}_{\text{0}}}^{*}=p_{{\text{x}}_{\text{0}}} \end{array}$$

(15-4) $$\begin{array}{l} L_{\text{min}}^{\text{x}}\leq p_{{\text{x}}_{\text{i}}}^{*}-p_{{\text{x}}_{\text{i}-\text{1}}}^{*}\leq L_{\text{max}}^{\text{x}},\;\text{i}=0...\text{k}-\text{1} \end{array}$$

Where *Q*_*x*_ is the weight for the robot's CoM position in the x-axis, aiming to minimize the error between the desired CoM position and the predicted position; *R*_*x*_ is the weight for the footstep placement, used to optimize the distance between the footstep placement of the i-th step and the (i−1)-th step. Equations 15-1, 2 represent the robot's CoM state prediction equation. Equation 15-4 represents the step boundary condition, preventing the foot placement from exceeding the reachable motion range of the swing leg. $L_{\text{min}}^{\text{x}}$ and $L_{\text{max}}^{\text{x}}$ represent the minimum and maximum boundary conditions, which are determined by the design of the robot's leg structure. Solving the above optimization problem allows for the determination of the optimal footstep placement along the x-axis for the future n-th step based on the current CoM state in the x-axis. Compared to optimization methods that rely on instantaneous velocity, this optimization approach uses the CoM position and footstep time to describe the desired average velocity, enabling the robot to accurately track the average velocity.

#### 2.2.2 y-z plane

During the y-axis motion of the LIP, the trajectory energy is typically less than zero, with both feet cyclically making contact with the ground on either side of the CoM. Based on the robot's desired velocity along the y-axis, ẏ_*des*_, and the footstep time for the future n-th step, $T_{\text{s}}^{n}$, the step length for the future n-th step can be estimated as:


(16) $$\begin{array}{l} W_{n}^{v}={ẏ}_{des}T_{s}^{n} \end{array}$$

The derivation method is similar to that in the x-z plane, and we can obtain the initial expectation and iterative formula as follows:


(17) $$\begin{array}{l} \left[\begin{array}{c} y_{com}^{0^{-}}\\ {ẏ}_{com}^{0^{-}} \end{array}\right]=Y_{0}^{-}=A\left(t_{e}^{0}-t_{0}\right)Y_{0}+B\left(t_{e}^{0}-t_{0}\right)p_{y_{0}} \end{array}$$

(18) $$\begin{array}{l} y_{com,des}^{1^{+}}=y_{com}^{0^{-}} \end{array}$$

(19) $$\begin{array}{l} y_{com,des}^{n^{+}}=y_{com,des}^{{\left(n-1\right)}^{-}}=y_{com,des}^{{\left(n-1\right)}^{+}}+W_{n-1}^{v} \end{array}$$

Where Equation 17 is used to calculate the robot's motion state at the end of the current support phase, and Equation 18 sets the motion state obtained from Equation 17 as the initial expected state for the future first step. The subsequent desired position along the y-axis can then be iteratively calculated using Equation 19. The illustration of the desired position along the y-axis is shown in Figure 4B. Thus, the objective function for the optimization problem in the y-z plane can be constructed as:


(20) $$\underset{P_{\text{x}}^{*}}{\min}\sum_{\text{i=1}}^{n}\left({\left\|y_{com,des}^{i^{+}}-y_{com}^{i^{+}}\right\|}_{Q_{y}}^{2}+{\left\|p_{y_{i}}^{*}-p_{w_{i}}\right\|}_{R_{y}}^{2}\right)$$

(20-1) $$\begin{array}{l} s\text{.}t\text{. }Y_{1}^{+}=A\left(t_{e}^{0}-t_{0}\right)Y_{0}+B\left(t_{e}^{0}-t_{0}\right)p_{y_{0}} \end{array}$$

(20-2) $${Y_{i}}^{+}=A\left(T_{\text{s}}^{i-\text{1}}\right){Y_{i-1}}^{+}B\left(T_{\text{s}}^{i-\text{1}}\right)p_{y_{i-1}}^{*}$$

(20-3) $$\begin{array}{l} p_{{\text{y}}_{\text{0}}}^{*}=p_{{\text{y}}_{\text{0}}} \end{array}$$

When the right foot is in contact with the ground:


(20-4) $$\begin{array}{l} L_{s}<{\left(-1\right)}^{i}\left(p_{x_{i}}^{*}-p_{x_{i-1}}^{*}\right)\leq L_{max}^{x} \end{array}$$

(20-5) $$\begin{array}{l} p_{w_{i}}=Y_{i,des}^{+}+{\left(-1\right)}^{i}W_{safe}\cosh\left(\frac{T_{s}\omega }{2}\right) \end{array}$$

When the left foot is in contact with the ground:


(20-6) $$\begin{array}{l} L_{s}<{\left(-1\right)}^{i-1}\left(p_{{\text{x}}_{\text{i}}}^{*}-p_{{\text{x}}_{\text{i}-\text{1}}}^{*}\right)\leq L_{\text{max}}^{\text{x}} \end{array}$$

(20-7) $$\begin{array}{l} p_{w_{i}}=Y_{i,des}^{+}+{\left(-1\right)}^{i-1}W_{safe}\cosh\left(\frac{T_{s}\omega }{2}\right) \end{array}$$

Where *Q*_*y*_ is the weight for the robot's CoM position in the y-axis; *p*_*w*_*i*__ represents the predicted footstep placement, derived from Equations 20-5, 7, which are used to constrain the footstep placement and prevent the distance between the foot placement in the y-z plane and the CoM position from becoming too short during walking; *R*_*y*_ is the weight for the foot placement position. Equations 20-1, 2 represent the robot's CoM state prediction equations along the y-axis. Equations 20-4, 6 define the boundary constraints for the reachable motion range of the swing leg. Unlike in the x-z plane, the optimization problem here introduces a safety margin *L*_*s*_ to prevent interference between the thigh or both feet. This parameter is related to the feasible motion range of the robot's design. *W*_*safe*_ is the set safety distance between the foot and the CoM when the velocity along the y-axis is zero.

Based on the optimization problem outlined above, the robot's optimal footstep placement in the X-Y plane can be obtained. The robot's motion follows the LIP dynamic model. Based on the desired footstep placement, the desired CoM acceleration can be calculated, and further integration yields the desired velocity and position.

## 3 Whole-body coordination controller

**Figure 5:**
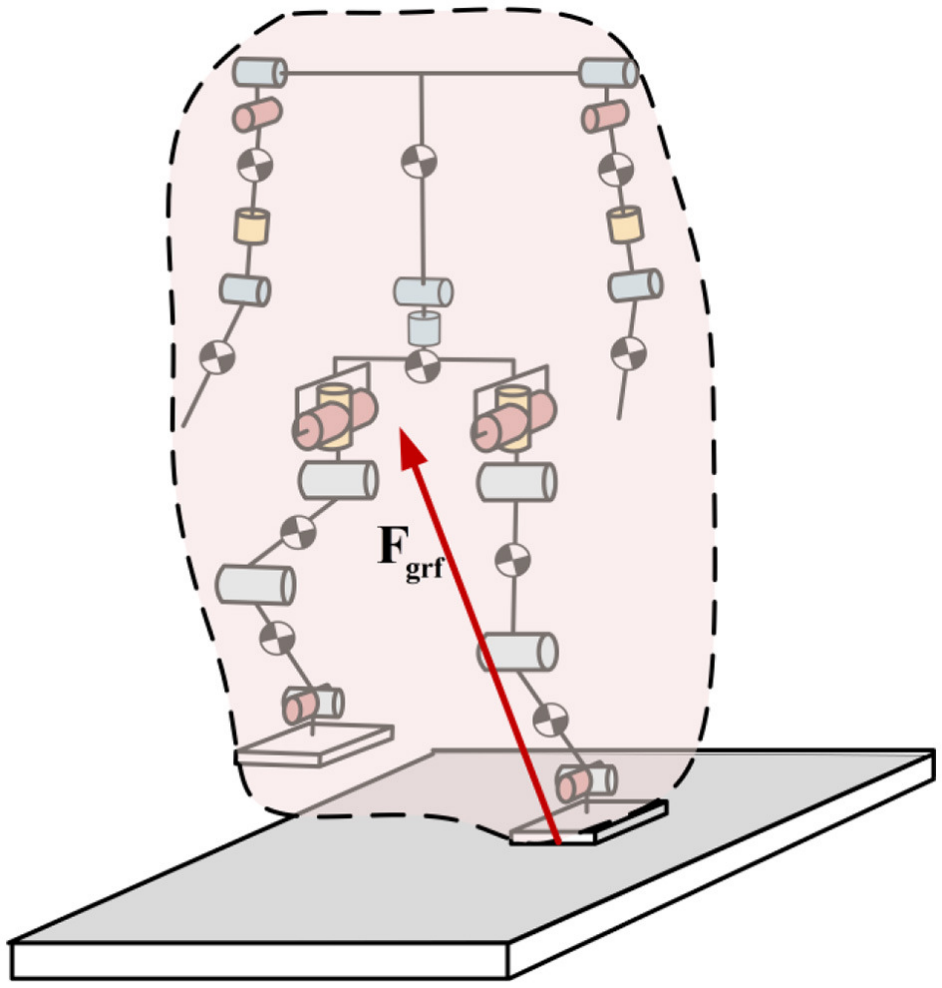
The contact force during the walking process of humanoid robot.

After obtaining the desired CoM trajectory for the robot, the primary task of the control system is to effectively track this trajectory. If we consider each frame during the walking process of a humanoid robot, the robot as a whole can be viewed as a large rigid body in each frame, as shown in Figure 5. The force driving this rigid body motion is the reaction force between the foot and the ground, which is applied only on the support plane formed by the supporting foot. The LIP model describes the relationship between the robot's supporting foot and the CoM. In the control systems of many bipedal robots, it can be observed that the system effectively controls the robot's CoM by coordinating the movement of the body. However, due to the limited degree of freedom configuration, the centroidal angle momentum generated during the swing foot's movement toward the desired position is difficult to control. As the robot's walking speed increases, the interference momentum caused by the leg swing becomes more pronounced, significantly affecting the stability of the robot's walking process. In biological studies related to human walking, it has been observed that the arms play a crucial role in maintaining stability during walking. From a robotics perspective, the arms do not need to interact with the external environment during walking, and they have a large and flexible range of motion. Therefore, how to effectively introduce coordinated arm swinging in the motion control system to counteract the disturbance momentum during dynamic walking is a key issue in enhancing the dynamic walking stability of the robot.

Furthermore, in existing research, the waist is typically used to enhance the degrees of freedom of the upper limbs for tasks such as grasping or transporting, while its role in walking is often overlooked. During walking, the waist serves as the pivotal link between the torso and the lower limbs, playing an essential role in driving the leg swing. However, its advantages in coordination with the lower limbs are seldom discussed. In related research, scholars have analyzed the advantages of utilizing the waist during humanoid robot walking from a kinematic perspective, proving that it can significantly increase the leg swing range, thereby benefiting the improvement of walking speed. However, this research is limited to the kinematic level. Therefore, the key issue in enhancing the robot's walking ability lies in how to effectively integrate waist motion into dynamic walking control, fully utilizing the active driving capability and kinematic advantages of the waist.

In traditional WBC, control tasks are introduced through methods like weighting or null-space projection, which demonstrate good control performance. As the robot's degrees of freedom and control tasks increase, traditional WBC becomes less effective due to the coupling of expected signals between multiple tasks. For instance, the waist joints are involved in various control tasks, such as robot body posture, swing foot position, foot contact position, and centroidal angle momentum. The coupling of the desired angles in the above tasks can lead to trade-offs in the waist joint between different tasks, making it difficult to achieve effective control. To address this issue, and in combination with the roles of the arms and waist during the walking process, a hierarchical framework-based whole-body coordination controller is proposed to control the dynamic walking of a humanoid robot.

### 3.1 Humanoid robot hierarchical dynamic modeling

Humanoid robot is a typical floating-base robotic system with a complex distribution of degrees of freedom. For the humanoid robot system used in this paper, each arm has 7 joints, each leg has 6 joints, and the waist has 2 joints. Considering the floating base, the robot has a total of 34 degrees of freedom. To prevent the coupling of multiple tasks during humanoid robot walking control from causing interference between joint control signals, a reasonable distribution of the robot's degrees of freedom and the tasks it performs is essential.

**Figure 6:**
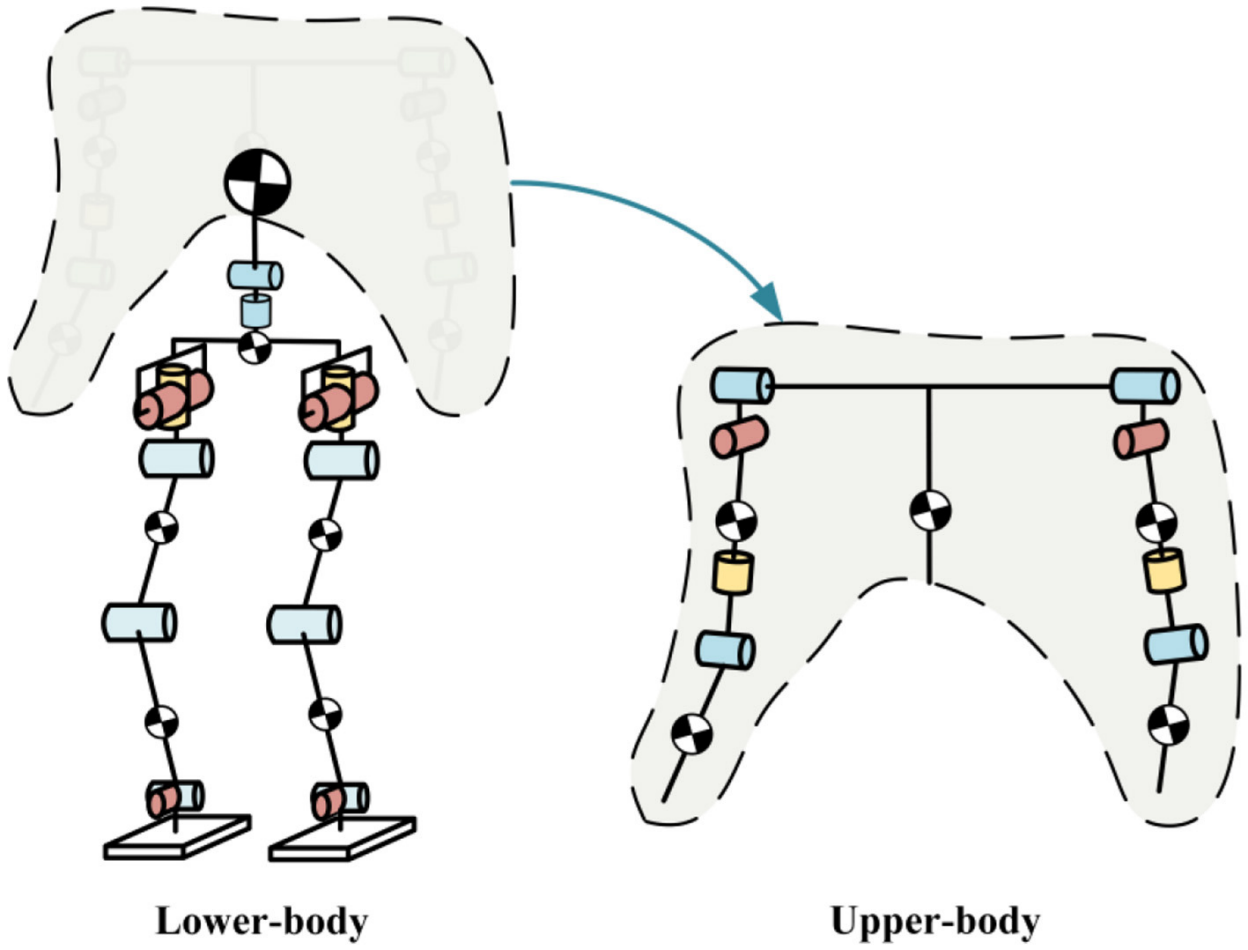
Hierarchical dynamic modeling of humanoid robot.

In this framework, we treat the torso and arms as the Upper-body of the robot, while the waist and legs are considered the Lower-body, as shown in Figure 6. According to the hierarchical principle, during the dynamic modeling of the floating base of the Lower-body, the entire Upper-body of the robot is treated as a large rigid body. The mass of this rigid body is the total mass of all the structures in the Upper-body, while the moment of inertia and CoM position are defined by the CoM position of the Upper-body at the initial state and the moment of inertia at that position. The specific dynamic model is as follows:


(21) $$\begin{array}{l} M_{d}\left(q_{d}\right){\ddot{q}}_{d}+C_{d}\left(q_{d},{\dot{q}}_{d}\right){\dot{q}}_{d}+G_{d}\left(q_{d}\right)=B_{d}\tau_{d}+J_{\text{c}}^{T}\text{λ} \end{array}$$

(22) $$\begin{array}{l} q_{d}={\left[\begin{array}{cc} q_{{\text{f}}_{d}}^{T} & q_{{\text{j}}_{d}}^{T} \end{array}\right]}^{T} \end{array}$$

Where $q_{{\text{f}}_{d}}\in {\mathbb{R}}^{6}$ represents the pose of the floating base in the world coordinate system, $q_{{\text{j}}_{d}}\in {\mathbb{R}}^{N_{d}}$ is the generalized position of the *N*_*d*_ driving joints in the Lower-body, $M_{d}\in {\mathbb{R}}^{\left(N_{d}+6\right)*\left(N_{d}+6\right)}$ is the inertia matrix of the Lower-body, $C_{d}\in {\mathbb{R}}^{\left(N_{d}+6\right)\times \left(N_{d}+6\right)}$ is the matrix accounting for the Coriolis and centrifugal effects in the Lower-body, $G_{d}\in {\mathbb{R}}^{N_{d}+6}$ is the gravitational term of the Lower-body, $B_{d}\in {\mathbb{R}}^{\left(N_{d}+6\right)\times N_{d}}$ is the selection matrix for the driving degrees of freedom in the Lower-body, $\tau_{d}\in {\mathbb{R}}^{N_{d}}$ represents the torque vector of the driving joints in the Lower-body, $\text{λ}\in {\mathbb{R}}^{6\times n_{\text{c}}}$ is a vector of stacked contact wrenches, $J_{\text{c}}\in {\mathbb{R}}^{\left(6\times n_{\text{c}}\right)\times \left(N_{d}+6\right)}$ is a matrix of stacked contact Jacobians, and *n*_*c*_ is the number of contact feet with the ground.

The Upper-body is modeled separately with its own dynamic model, which is as follows:


(23) $$\begin{array}{l} M_{u}\left(q_{u}\right){\ddot{q}}_{u}+C_{u}\left(q_{u},{\dot{q}}_{u}\right){\dot{q}}_{u}+G_{u}\left(q_{u}\right)=B_{u}\tau_{u} \end{array}$$

(24) $$\begin{array}{l} q_{u}={\left[\begin{array}{cc} q_{{\text{f}}_{u}}^{T} & q_{{\text{j}}_{u}}^{T} \end{array}\right]}^{T} \end{array}$$

In this stage of modeling, we choose the torso as the base. Unlike the floating base dynamic modeling used for the Lower-body, there are no external contact forces involved in the Upper-body modeling. However, in contrast to traditional fixed base modeling, the Upper-body model continuously provides the posture and position of the base through state estimation data during the robot's movement. Where $q_{{\text{f}}_{u}}\in {\mathbb{R}}^{6}$ represents the pose of the base in the world coordinate system, $q_{{\text{j}}_{u}}\in {\mathbb{R}}^{N_{u}}$ is the generalized position of the *N*_*u*_ driving joints in the Upper-body, $M_{u}\in {\mathbb{R}}^{\left(N_{u}+6\right)*\left(N_{u}+6\right)}$ is the inertia matrix corresponding to the Upper-body, $C_{u}\in {\mathbb{R}}^{\left(N_{u}+6\right)\times \left(N_{u}+6\right)}$ is the matrix accounting for the Coriolis and centrifugal effects in the Upper-body, $G_{u}\in {\mathbb{R}}^{N_{u}+6}$ is the gravitational term of the Upper-body, $B_{u}\in {\mathbb{R}}^{\left(N_{u}+6\right)\times N_{u}}$ is the selection matrix for the driving degrees of freedom in the Upper-body, and $\tau_{u}\in {\mathbb{R}}^{N_{u}}$ is the torque vector of the driving joints in the Upper-body.

In the above two dynamic models, the kinematic and dynamic parameters can be obtained through measurement and identification methods. The robot's components can be simplified as equivalent links, and the centroid coordinates of each link can be obtained using the D-H transformation. The CoM position can be calculated from the centroid positions of each link.


(25) $$\begin{array}{l} r_{c}=\frac{m_{1}p_{com}^{1}+m_{2}p_{com}^{2}...+m_{n_{L}}p_{com}^{n_{L}}}{m_{1}+m_{2}...+m_{n_{L}}} \end{array}$$

Where *r*_*c*_ represents the robot's CoM coordinates, *n*_*L*_ is the number of equivalent links in the humanoid robot, $p_{com}^{n_{L}}$ is the CoM coordinates of component *n*_*L*_, and *m*_*n*_*L*__ is the mass of component *n*_*L*_. The robot's centroid Jacobian matrix *J*_com_ can be derived based on the CoM position formula. The centroidal angle momentum can be derived using the following equation:


(26) $$\begin{array}{l} h_{c}=A_{c}\left(q\right)\dot{q} \end{array}$$

Where $h_{c}\in {\mathbb{R}}^{6}$ represents the centroidal momentum. *A*_*c*_ is the centroidal momentum matrix, which describes the contribution of each link and the floating base to the centroidal momentum.

### 3.2 Lower-body WBC

The Lower-body is responsible for maintaining contact with the ground and supporting the robot's walking during the walking process. Unlike most existing control frameworks, we introduce a two-degree-of-freedom waist joint in our framework. Based on this, we list the Lower-body WBC tasks as shown in Table 1.

**Table 1:** Lower-body WBC tasks.

	**Task**
1	CoM position
2	Swing foot pose
3	Torso posture
4	Waist joint angle

The CoM position and swing leg pose tasks are the focus of the Lower-body WBC. Its control accuracy will directly determine the robot's ability to track the planned footstep placement and CoM trajectory described in Section 2. The torso posture task ensures that the robot can effectively adjust its torso angle to prevent excessive deviation and tipping over. The waist joint angle task is assigned the lowest priority, allowing the robot's pelvis to move flexibly according to higher-priority tasks. Based on the above description, the optimization problem for constructing the Lower-body WBC is formulated as follows:


(27) $$\underset{{\ddot{q}}_{d}^{cmd},\text{λ},\tau }{\min}\sum_{\text{i}=1}^{e}\left({\left\|J_{i}^{d}{\ddot{q}}_{d}^{cmd}+{\dot{J}}_{i}^{d}{\dot{q}}_{d}-{\dot{w}}_{\text{d},\text{ i}}\right\|}_{Q_{w}}^{2}\right)+{\left\|{\ddot{q}}_{d}^{cmd}\right\|}_{Q_{a}}^{2}$$

(27-1) $$\begin{array}{l} M_{d}{\ddot{q}}_{d}^{cmd}+C_{d}{\dot{q}}_{d}+G_{d}=B_{d}\tau_{d}+J_{\text{c}}^{T}\text{λ} \end{array}$$

(27-2) $$\begin{array}{l} J_{\text{c}}{\ddot{q}}_{d}^{cmd}+{\dot{J}}_{\text{c}}{\dot{q}}_{d}=0 \end{array}$$

(27-3) $$\begin{array}{l} A_{f}\text{λ}\leq 0 \end{array}$$

(27-4) $$\begin{array}{l} A_{\text{zmp}}\text{λ}\leq 0 \end{array}$$

(27-5) $$\begin{array}{l} 0\leq S\text{λ}\leq F_{\text{c},\text{z}}^{\text{max}} \end{array}$$

(27-6) $$\begin{array}{l} I_{min}\left(q_{d},{\dot{q}}_{d}\right)\leq {\ddot{q}}_{d}^{cmd}\leq I_{max}\left(q_{d},{\dot{q}}_{d}\right) \end{array}$$

(27-7) $$\begin{array}{l} \tau_{\min}\leq \tau \leq \tau_{\max} \end{array}$$

**Cost function:** the cost function is divided into two parts. The first part is the summation of the cost functions for each designed task, which aims to control the robot's effective tracking of the desired tasks. Here, *e* is the total number of tasks, ${\text{J}}_{i}^{d}$ is the Jacobian matrix of task i in the task space, ẇ_d, i_ is the target task acceleration command, and *Q*_*w*_ is the task execution weight. ẇ_d, i_ is the task execution acceleration, which is obtained through the desired acceleration and a PD controller.


(28) $$\begin{array}{l} {ẇ}_{\text{d, i}}={ẇ}_{\text{d, i}}^{\text{des}}+K_{\text{p}}^{\text{i}}\left(x_{\text{d, i}}^{\text{des}}-x_{\text{d, i}}\right)+K_{\text{d}}^{\text{i}}\left(w_{\text{d, i}}^{\text{des}}-w_{\text{d, i}}\right) \end{array}$$

Where $x_{\text{d, i}}^{\text{des}}$, $w_{\text{d, i}}^{\text{des}}$, and ${ẇ}_{\text{d, i}}^{\text{des}}$ represent the desired position, velocity, and acceleration of the i-th task for the Lower-body, respectively; *x*_d, i_ and *w*_d, i_ represent the actual position and velocity of the i-th task; $K_{\text{p}}^{\text{i}}$ and $K_{\text{d}}^{\text{i}}$ are the proportional and derivative feedback coefficients for the i-th task. In this optimization problem, we can adjust the priority of multiple tasks by tuning the weight parameters, and integrate the desired angular acceleration for each task, thereby obtaining the optimal motion trajectory for the robot.

The second part of the cost function aims to minimize the acceleration of the driving joints in order to fully activate the waist's role during the walking process. This allows the waist to seek the optimal coordinated motion trajectory during the stance and swing leg movements, thereby maximizing the robot's maximum stride length.

**Dynamic constraints:** the Equation 21 is used as the constraint (Equation 27-1) in the optimization problem, ensuring that the optimization results satisfy the floating base dynamic equation of the lower body constructed in Section 3.1.

**Contact constraints:** during the walking process of the humanoid robot, we assume that the foot in contact with the ground does not experience relative displacement with respect to the ground. Therefore, constraint (Equation 27-2) is defined to restrict the robot's motion.

**Figure 7:**
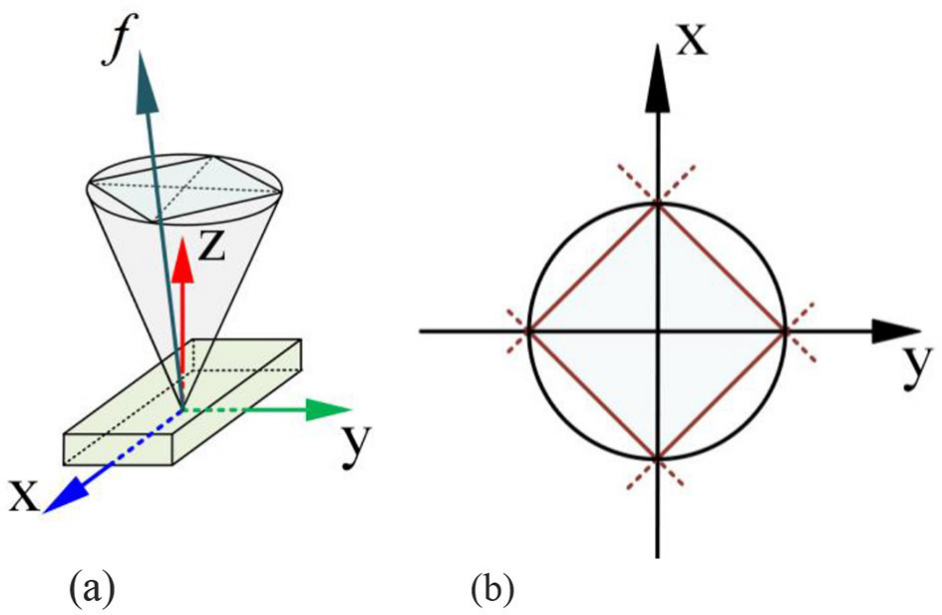
Illustration of the foot contact friction constraints. **(A)** 3-D space. **(B)** 2-D space projection.

**Friction constraints:** in constraint (Equation 27-2), we assume that the foot in contact with the ground does not experience relative displacement with respect to the ground. From the perspective of contact mechanics, the contact force at the foot must adhere to friction constraints. These friction constraints can be approximated by a pyramid, as shown in Figure 7. In this optimization problem, the friction constraint is given by Equation 27-3, where *A*_*f*_ is the friction constraint matrix.

**Contact foot ZMP constraint**: the ZMP is the point where the horizontal component of the robot's foot contact force and moment balance are zero on a flat surface. To prevent the robot's foot from tipping due to excessive torque, we set constraint (Equation 27-4) to ensure that the robot's ZMP remains within the support region of the robot's foot. Here, **A**_*zmp*_ is the ZMP constraint matrix.

**Unilateral contact force constraint**: during walking, the foot is subject to unilateral contact forces from the ground. The constraint is given by Equation 27-5, where *S* is the selection matrix and $F_{c,z}^{\max}$ is the maximum contact force in the z-axis at the foot.

**Joint motion constraints:** constraint (Equation 27-6) is imposed to restrict joint accelerations, preventing the robot's joints from exceeding position or velocity limits.

**Joint torque constraints:** constraint (Equation 27-7) is applied to limit the joint driving torques, where τ_min_ and τ_max_ represent the minimum and maximum torque vectors for the driving joints, respectively.

Through the above optimization problem, we can obtain the optimal driving acceleration, driving torque for each Lower-body joint, and the optimal contact force between the foot and the ground. The desired joint positions $q_{d}^{cmd}$ and velocities ${\dot{q}}_{d}^{cmd}$ can be obtained through the integration of the accelerations. The centroidal momentum generated during the walking process can be estimated using the desired joint angles and velocities of the Lower-body joints:


(29) $$\begin{array}{l} {h_{c}}^{*}=A_{c}^{d}\left({q_{d}}^{cmd}\right){{\dot{q}}_{d}}^{cmd} \end{array}$$

(30) $$\begin{array}{l} {{ḣ}_{c}}^{*}={Ȧ}_{c}^{d}\left({q_{d}}^{cmd}\right){{\dot{q}}_{d}}^{cmd}+A_{c}^{d}\left({q_{d}}^{cmd}\right){{\ddot{q}}_{d}}^{cmd} \end{array}$$

In the equation, ${h_{c}}^{*}$ represents the centroidal momentum calculated using the desired signals during the walking control of the Lower-body WBC, and ${{ḣ}_{c}}^{*}$ is the time derivative of the centroidal momentum. To further align with the ideal LIP model, the desired centroidal angle momentum during the walking process is zero. We will compensate for this by utilizing the swing of the arms, driven by the Upper-body WBC.

### 3.3 Upper-body WBC

Compared to the Lower-body WBC, which interacts with the environment during walking tasks, the primary task of the Upper-body is to drive the swinging of arms to enhance the stability of the robot during walking. Based on this, we list the Upper-body WBC tasks as shown in Table 2.

**Table 2:** Upper-body WBC tasks.

	**Task**
1	CoM position
2	Centroidal angle momentum
3	Swing hand pose

In the Lower-body dynamics modeling process in Section 3.1, we set the overall CoM position of the Upper-body. Therefore, one of the most important tasks in the Upper-body WBC is to control the set CoM position, as failure to do so will affect the estimation of the overall CoM trajectory during the control process. Task 2 is to compensate for the centroidal angular momentum deviation ${h_{c}}^{*}$ caused by the Lower-body WBC during the walking control by controlling the arm's angular momentum. Compared to some existing studies that design arm swinging to counteract the CoM's yaw-axis angular momentum, we independently construct a controller to simultaneously compensate for the angular momentum along all three axes of the centroid. Task 3 is to design a default swinging trajectory for the robot's arm end, and this task is assigned the lowest priority to maintain the arm's regular swinging motion. Based on the above description, the optimization problem for the Upper-body WBC is constructed as follows:


(31) $$\underset{{\ddot{q}}_{u},\tau }{\min}\sum_{i=1}^{u}\left({\left\|J_{i}^{u}{\ddot{q}}_{u}^{cmd}+J_{i}^{u}{\dot{q}}_{u}-{\dot{w}}_{u,\;i}\right\|}_{Q_{v}}^{2}\right)$$

(31-1) $$\begin{array}{l} M_{u}{\ddot{q}}_{u}+C_{u}{\dot{q}}_{u}+G_{u}=B_{u}\tau_{u} \end{array}$$

(31-2) $$\begin{array}{l} B\left(q_{u},{\dot{q}}_{u},{\ddot{q}}_{u}^{cmd}\right)<0 \end{array}$$

(31-3) $$\begin{array}{l} I_{\min}\left(q_{u},{\dot{q}}_{u}\right)\leq {\ddot{q}}_{u}\leq I_{\max}\left(q_{u},{\dot{q}}_{u}\right) \end{array}$$

(31-4) $$\begin{array}{l} \tau_{\min}\leq \tau \leq \tau_{\max} \end{array}$$

**Cost function:** the cost function is relatively simple. $J_{i}^{u}$ is the Jacobian matrix for the i-th task in the task space, ẇ_u, i_ is the target task acceleration command, and *Q*_*v*_ is the task execution weight. By adjusting the weight parameters, the priorities of multiple tasks are adjusted, and the expected angular accelerations of the tasks are integrated to derive the optimal motion trajectory for the robot. Tasks 1 and 3 are constructed similarly to the Lpper-body WBC. The feedback function for Task 2 can be formulated as:


(32) $${\dot{w}}_{\text{u,2}}=-{\dot{h}}_{c}^{*}-K_{h}h_{c}^{*}$$

Task 2's Jacobian matrix is the same as the angular momentum matrix:


(33) $$\begin{array}{l} J_{2}^{u}=A_{c}^{u}\left(q_{u}\right) \end{array}$$

Based on the task setup above, the arm swinging can compensate for the angular momentum generated during the Lower-body control process.

**Dynamics constraints**: the Equation 23 is used as a constraint (Equation 31-1) in the optimization problem to ensure the results satisfy the Upper-body dynamics model presented in Section 3.1.

**Arm swing collision constraint**: upon observation of the robot's arm swinging process, it is found that when the forearm does not collide with the body, the entire arm will not collide with the body. Therefore, the forearm is selected as the constraint target. Four marker points are placed on both left and right forearms. The positions, velocities, and accelerations of these points are computed using kinematics. The boundary of the body shape is set as the boundary condition for the marker point motion, creating inequality constraints to prevent collisions between the robot's arms and body during integration of position and velocity.

**Joint motion constraints:** constraint (Equation 31-3) is set to limit the joint acceleration values to prevent the robot's joints from exceeding position or velocity limits.

**Joint torque constraints:** constraint (Equation 31-4) is set to limit the joint torques, where τ_min_ and τ_max_ are the minimum and maximum torque vectors for the driving joints, respectively.

## 4 Simulation experiments

### 4.1 Software framework and robot platform

The humanoid robot control system is primarily developed using a C++ multithreading framework. Control algorithms are constructed with the Drake robotics library, and the robot's dynamics and motion parameters are described uniformly using URDF files. For solving optimization problems within the control system, the SNOPT solver is called through the API in Drake. Additionally, C++ is integrated with RaiSim to communication, serving as the simulation environment and physics engine for robot walking control.

**Figure 8:**
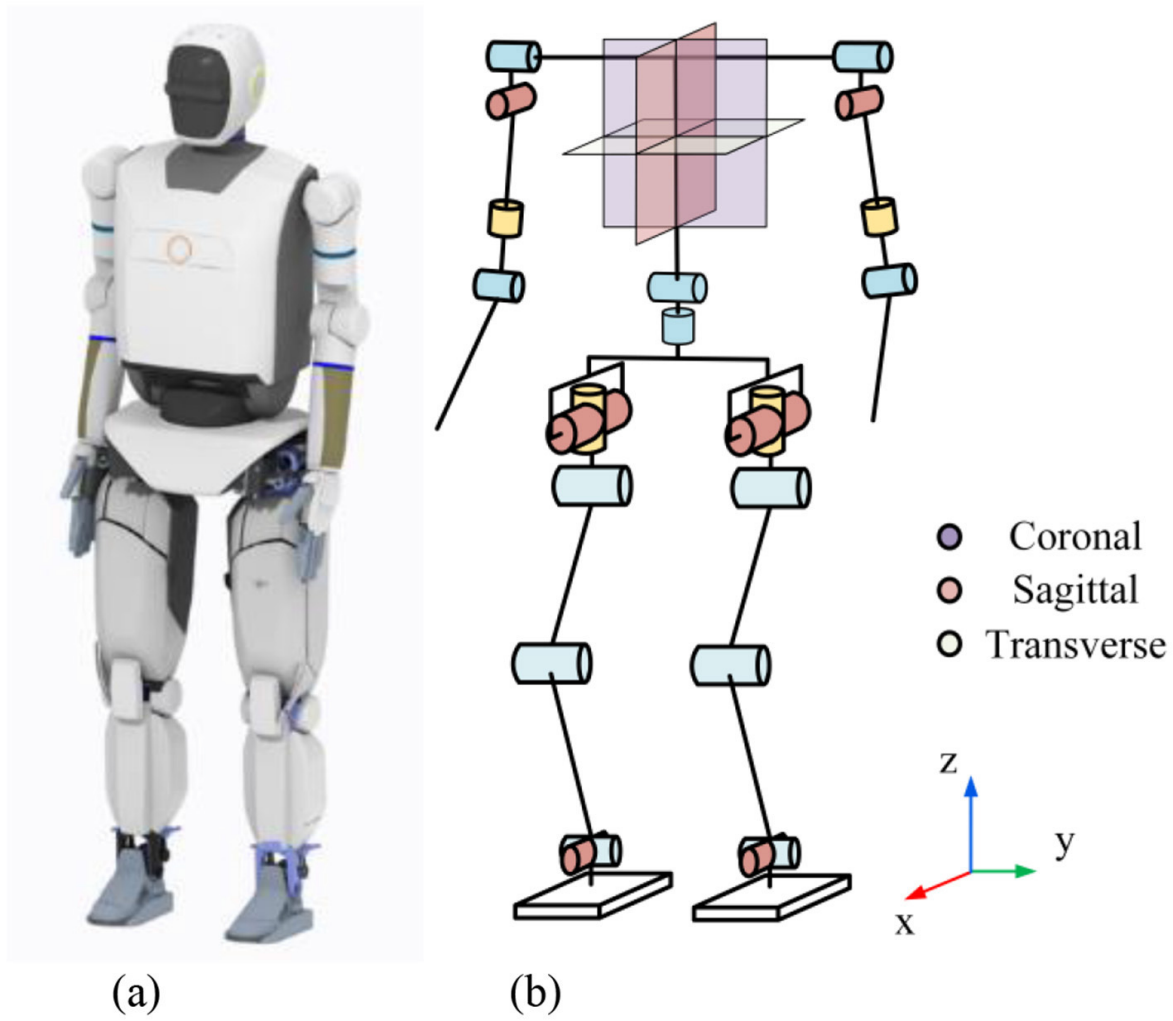
The Dexbot humanoid robot simulation prototype. **(A)** Rendered image. **(B)** Robot joint distribution.

The robot used in the simulation is the Dexbot humanoid robot system, developed by Harbin Institute of Technology. The robot stands at a height of 175 cm and weighs approximately 58 kg. It has a total of 28 active joints, excluding the dexterous hands. Each leg features 6 joints, the waist has 2 joints, and each arm has 7 joints. Since the three degrees of freedom at the arm's end mainly control hand posture and do not participate in the walking process, each arm is simplified to 4 degrees of freedom for simulation. The robot and its degree-of-freedom distribution is shown in Figure 8. The robot's innovative humanoid design ensures efficient energy utilization, and its high power-to-weight ratio custom electric actuators enable high dynamic movement potential. For clarity in the simulation, all external body casing is removed. The robot's mass of links is listed in Table 3. The joint limit angles are also provided in Table 4.

**Table 3:** Mass of links.

**Rigid part**	**Mass (kg)**
Torso	22.7
Pelvic	8.05
Thigh	5.6
Shank	4.2
Foot	1.01
Upper arm	1.82
Forearm	1.57

**Table 4:** Joint limit angles.

**Joint name**	**Range of motion**
Hip roll	−0.34-0.26
Hip pitch	−1.29-0.26
Hip yaw	−0.17-0.17
Knee	0-1.92
Ankle pitch	−1.1-0.43
Ankle roll	−0.29-0.29

### 4.2 CoM trajectory numerical simulation

**Figure 9:**
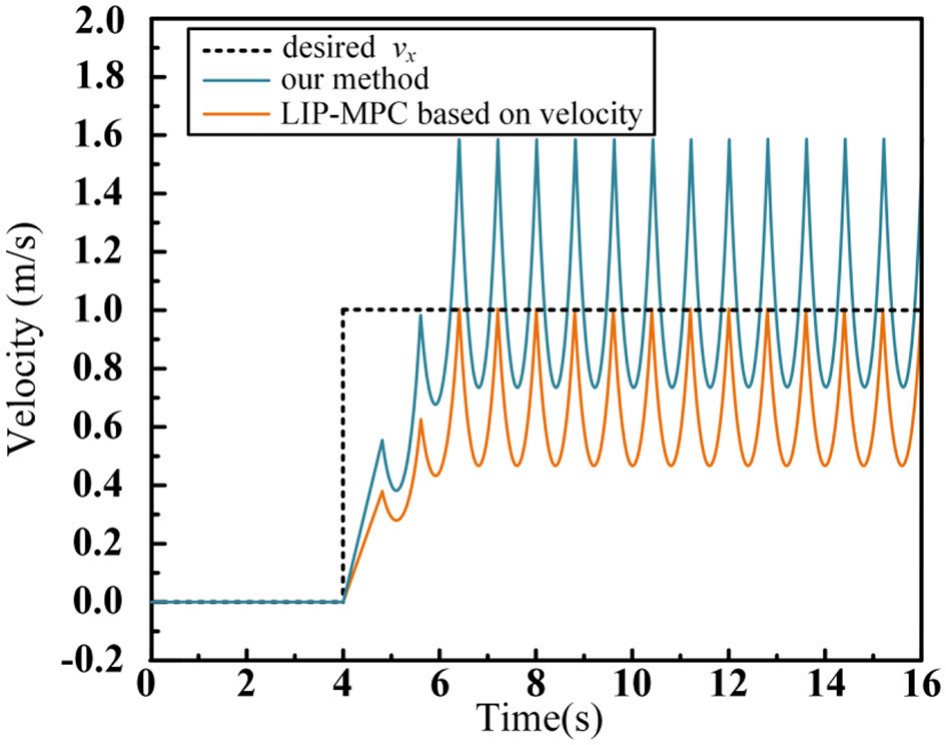
Average velocity tracking data curve in numerical simulation.

To validate the improved footstep planner proposed in Section 2 of this paper, we designed a numerical simulation to visually demonstrate the advantages of our method over traditional approaches. The input to the numerical simulation is a step input of the desired walking velocity. At the 4th second of the simulation, the desired velocity changes from 0 m/s to 1 m/s, with a footstep time T_s_ = 0.8. The simulation results are shown in Figure 9.

In the simulation, we compare the traditional LIP-MPC method with the footstep planning method proposed in this paper. Traditional methods often use the instantaneous velocity at the end of each walking phase as the prediction model for the MPC, which only ensures that the velocity at the final time of each step reaches the desired speed, but fails to guarantee the average velocity during the entire support phase. As shown in the Figure 9, after the motion reaches a stable state, the average velocity of traditional LIP-MPC is 0.632 m/s, with a tracking error of 0.368 m/s, resulting in a tracking accuracy of only 63.2%. In contrast, the method proposed in this paper avoids using the instantaneous velocity formula for predicting the centroid motion. Instead, we derive and iterate the displacement over a single support phase, which ensures proper displacement of the centroid within each cycle and consequently achieves good average velocity control performance. In the numerical simulation, our method exhibits no tracking error.

The improved footstep planner proposed in Section 2 of this paper effectively eliminates tracking errors in the planning phase of the desired velocity, thereby enhancing the accuracy of the robot's actual speed tracking during the walking process.

### 4.3 Acceleration walking control simulation

To validate the improvement in the humanoid robot's maximum walking ability and dynamic stability using the walking control framework proposed in this paper, three conditions were designed for comparison: (1) no arm and waist swinging, (2) with waist swinging, and (3) with both waist and arms swinging. Walking data for each condition were compared and analyzed. During the walking process, the footstep time T_s_ = 0.8s. A walking command was sent to the robot at the 10th second, and the desired walking speed was incremented by 0.15 m/s every subsequent 10 seconds. The simulation snapshots and data for each condition are shown below.

(1) No arm and waist swinging

In this experiment, the footstep planner proposed in Section 2 and the lower-body WBC (with the waist control part temporarily disabled) were used to track and control the desired walking speed input by user. For this condition, the humanoid robot's waist joints and arms were set to fixed angles and did not participate in the walking process.

**Figure 10:**
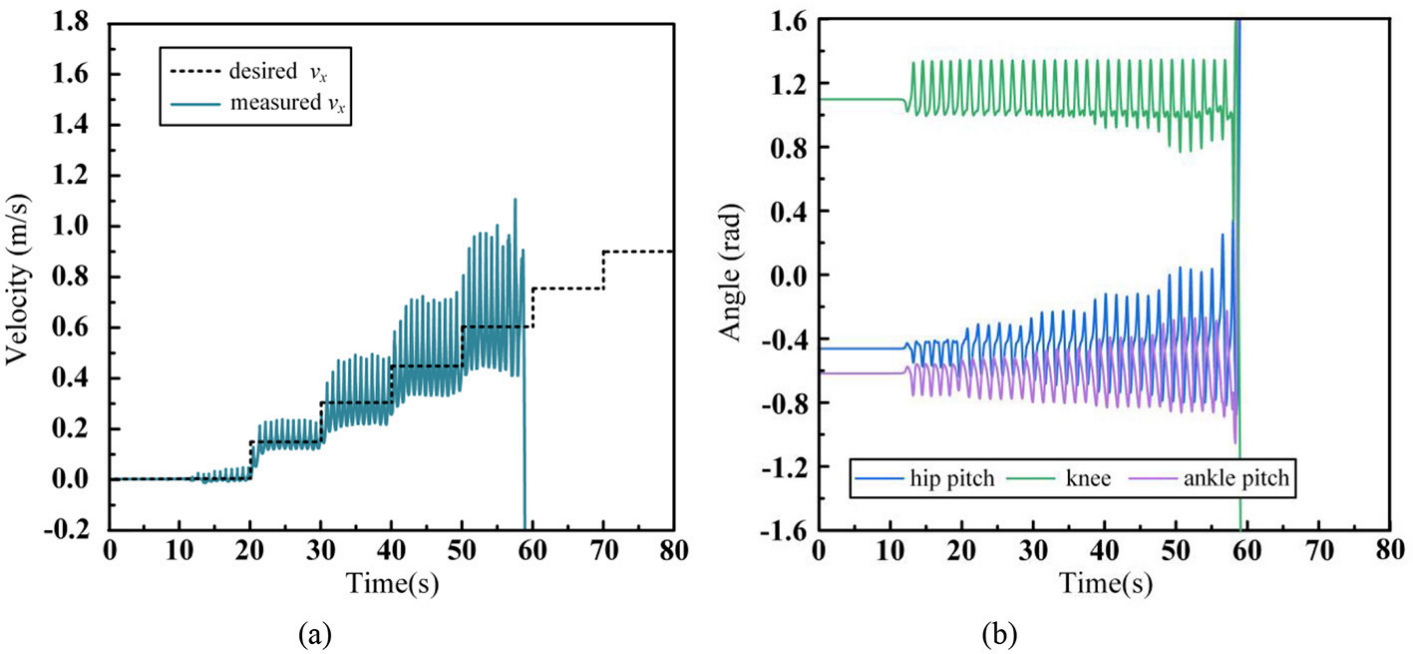
Humanoid robot acceleration walking data without arm and waist swinging. **(A)** CoM velocity. **(B)** Leg joint angles.

In Figure 10A shows the tracking performance of the robot's CoM with respect to the desired walking speed, Figure 10B displays the joint angles of the left leg during the walking process. From Figure 10A, it is evident that the humanoid robot starts from 0 m/s and accelerates stably to 0.45 m/s with each step increase of 0.15 m/s, achieving stable tracking of the desired speed. However, when the robot accelerates from 0.45 m/s to 0.6 m/s, the robot initially achieves stable acceleration and quickly reaches effective tracking of the desired speed. But as the walking process continues, the robot struggles to control its speed effectively, leading to a fall. Referring to Figure 10B, we observe that as the robot's walking speed increases, the range of motion for the hip, knee, and ankle joints also increases. When the speed reaches 0.6 m/s, the knee joint's range of motion is approximately 0.6 to 1.3 rad, the hip pitch angle ranges from −0.83 to 0.2 rad, and the ankle pitch angle ranges from −0.88 to −0.17 rad. These motion ranges are close to the limits of the robot's joint movement. Additionally, the robot's instability at an average walking speed of 0.6 m/s causes significant speed fluctuations, and the footstep positions need frequent large adjustments to control the robot's speed. In summary, during dynamic walking, the insufficient margin for joint movement, combined with large adjustments in the swinging leg, leads to joint overextension, eventually causing the robot to fall.

From this scenario, it can be observed that, with a fixed footstep time T_s_, the maximum step length is a key parameter influencing the robot's maximum walking speed. The longer the maximum step length, the greater the effective working range for both the swinging and supporting legs, which increases the range of adjustments that can be made during walking. At the same time, enhances the robot's ability to handle external disturbances such as the impact force between the foot and the ground, as well as inertial forces.

(2) With waist swing

**Figure 11:**
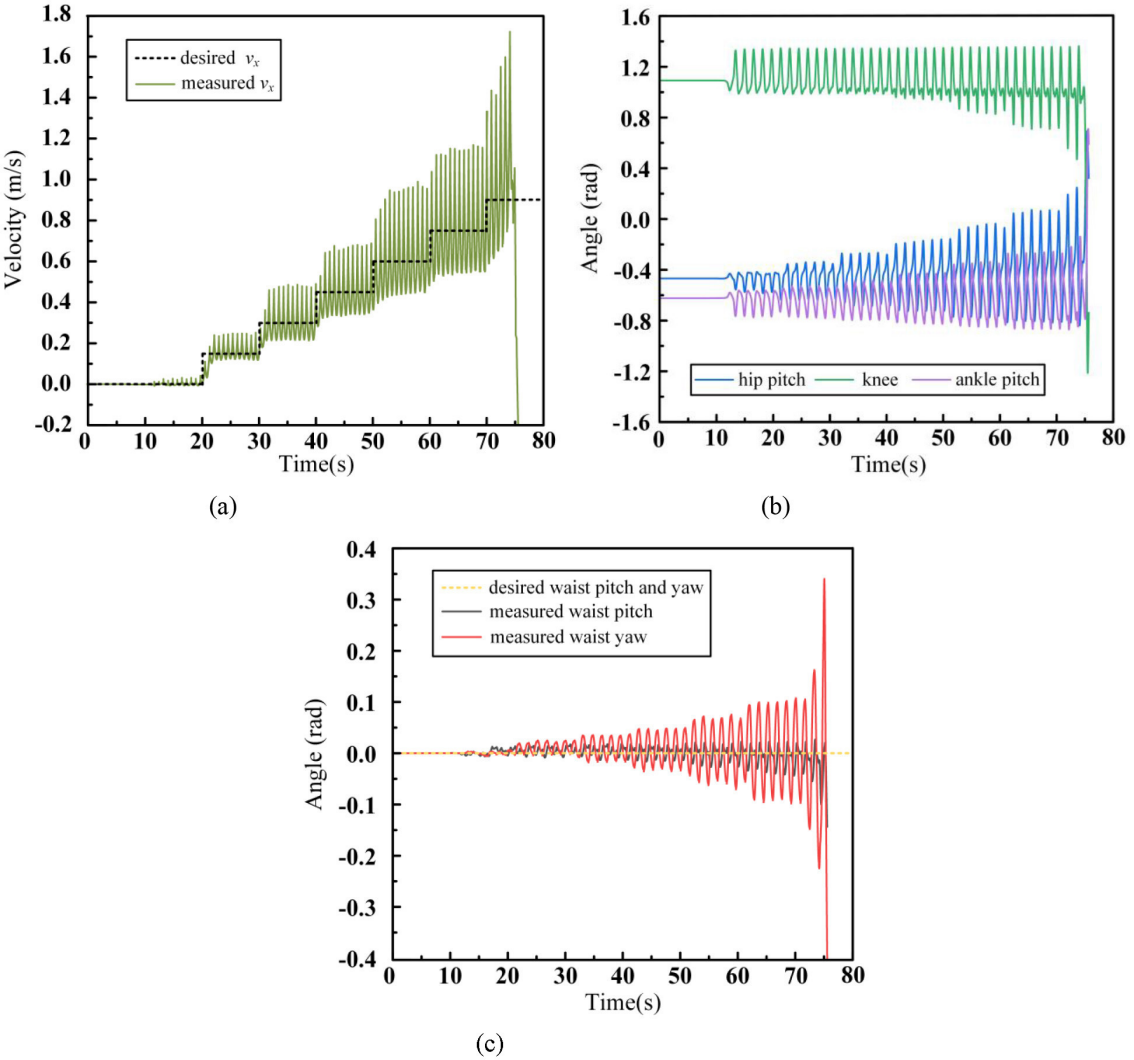
Humanoid robot acceleration walking data with waist swinging. **(A)** CoM velocity. **(B)** Leg joint angles. **(C)** Waist joint angles.

Based on the conclusions from scenario (1) and the analysis in Section 3 of the paper, introducing waist motion can increase the effective movement range of the robot's foot, thereby enhancing the robot's maximum walking speed. Therefore, in this scenario, waist control is introduced compared to scenario (1) to verify the advantages and effectiveness of incorporating waist motion into the Lower-body WBC in the hierarchical coordination controller proposed in this paper. The walking control data for the humanoid robot with waist swing is as follows:

In Figure 11A shows the robot's CoM tracking of the desired walking speed, Figure 11B represents the angle values of key joints in the left leg during walking, and Figure 11C shows the motion trajectory of the waist joints during walking. From Figure 10A, it can be seen that the humanoid robot accelerates steadily from 0 m/s to 0.75 m/s by increasing the desired speed by 0.15 m/s at each step and achieves stable tracking. However, it falls when further accelerated to 0.9 m/s. Compared to scenario (1), where waist control was not introduced, the robot's stable tracking speed improved from 0.45 m/s to 0.75 m/s, increasing the robot's maximum walking speed by 0.3 m/s. Referring to the waist motion trajectory in Figure 10C, it can be observed that as the walking speed increases, the robot's waist Yaw angle swing increases, while the pitch angle is used to coordinate the pelvis and torso posture, thus better utilizing the Yaw angle to contribute to the walking stride length. Comparing Figure 11B with Figure 10B from scenario (1), it can be seen that the robot's leg motion range is smaller at the same speed. This is because the waist joints, through the rotation of the pelvis, significantly increase the movement boundaries of the robot's foot, allowing it to cover a greater distance with a smaller leg swing range. This directly proves that the coordinated waist swing can increase the humanoid robot's maximum stride length during walking, thereby enhancing its walking capabilities.

Based on this scenario, it can be observed that by introducing coordinated waist motion, the robot's maximum stride length can be increased while keeping the footstep time T_s_ constant. At the same walking speed, the robot's leg joint swing amplitudes are smaller, leaving more motion margin to maintain stable walking. However, when the robot is walking at higher speeds, its centroidal angle momentum increases rapidly. This causes difficulties in adjusting the robot's desired motion states effectively within the ZMP constraint, ultimately leading to walking failure. The humanoid robot driven by linear electric actuators, due to its design mimicking the distribution and force characteristics of human muscles, has a more dispersed mass. As a result, during high-speed walking, the momentum shift caused by the swing leg becomes particularly pronounced. In such cases, compensating for the center of mass momentum using arm swing is crucial.

(3) With waist and arms swing

**Figure 12:**
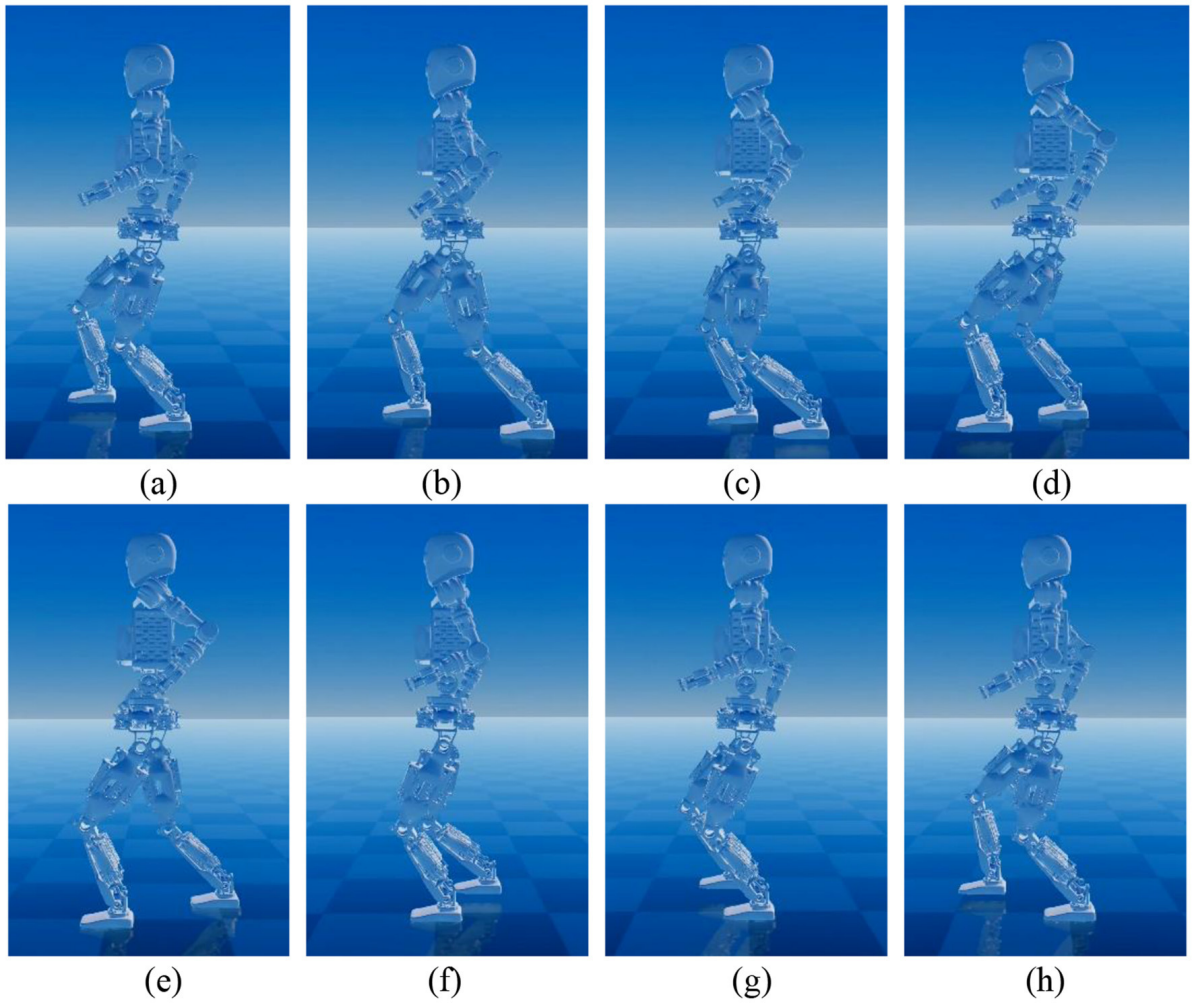
Humanoid robot acceleration walking snapshots with waist and arms swinging.

Building upon the conclusions from Scenario (2) and the analysis in Section 3 of this paper, the arm movement during walking is unconstrained, allowing for flexible swinging. Therefore, in this scenario, compared to Scenario (2), we introduce control for coordinated arm swing to validate the advantages and effectiveness of the upper body WBC in the proposed layered coordination controller. Simulation snapshots of the humanoid robot's walking process are shown in Figure 12.

**Figure 13:**
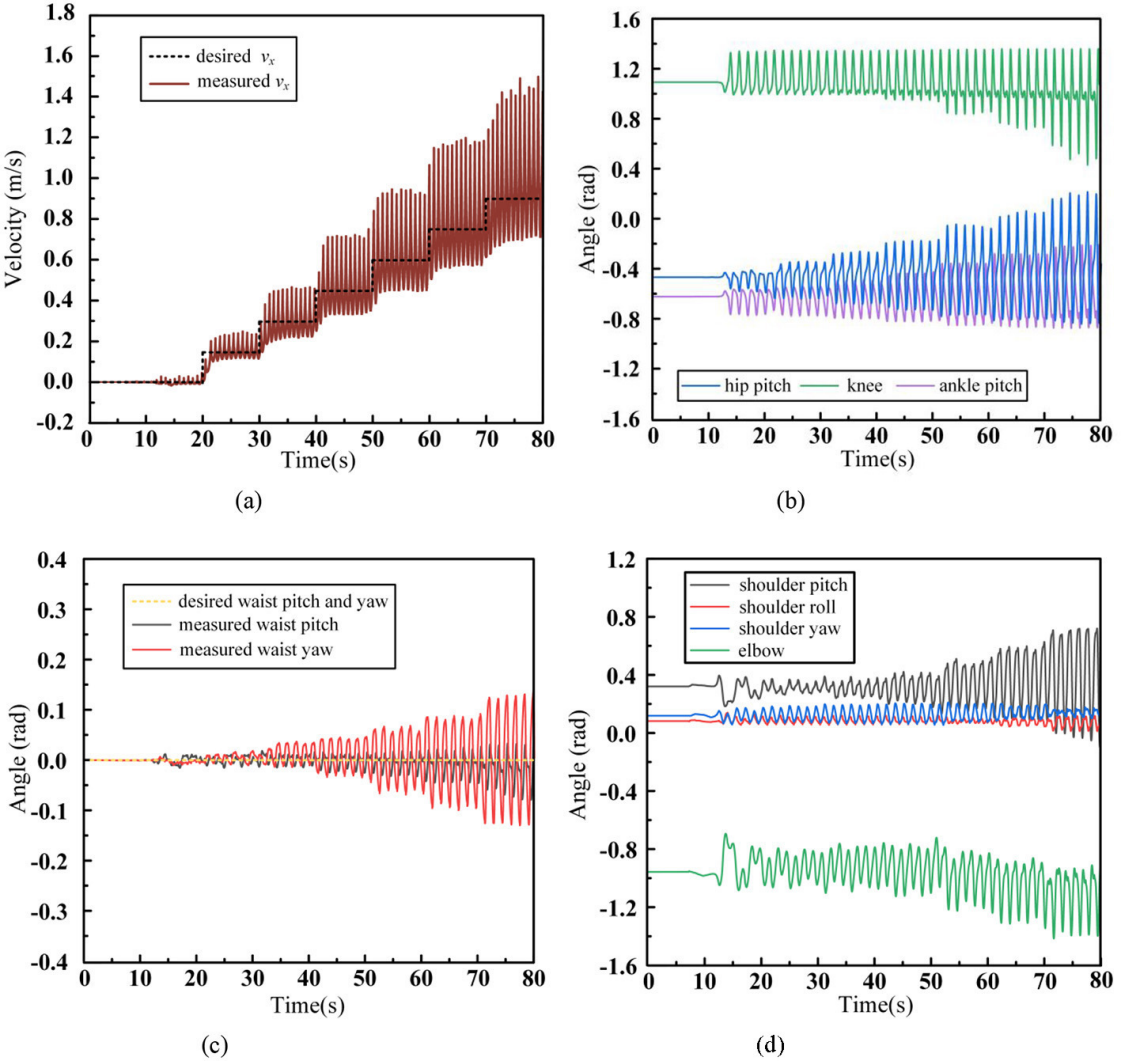
Humanoid robot acceleration walking data with waist and arms swinging. **(A)** CoM velocity. **(B)** Leg joint angles. **(C)** Waist joint angles. **(D)** Arm joint angles.

**Figure 14:**
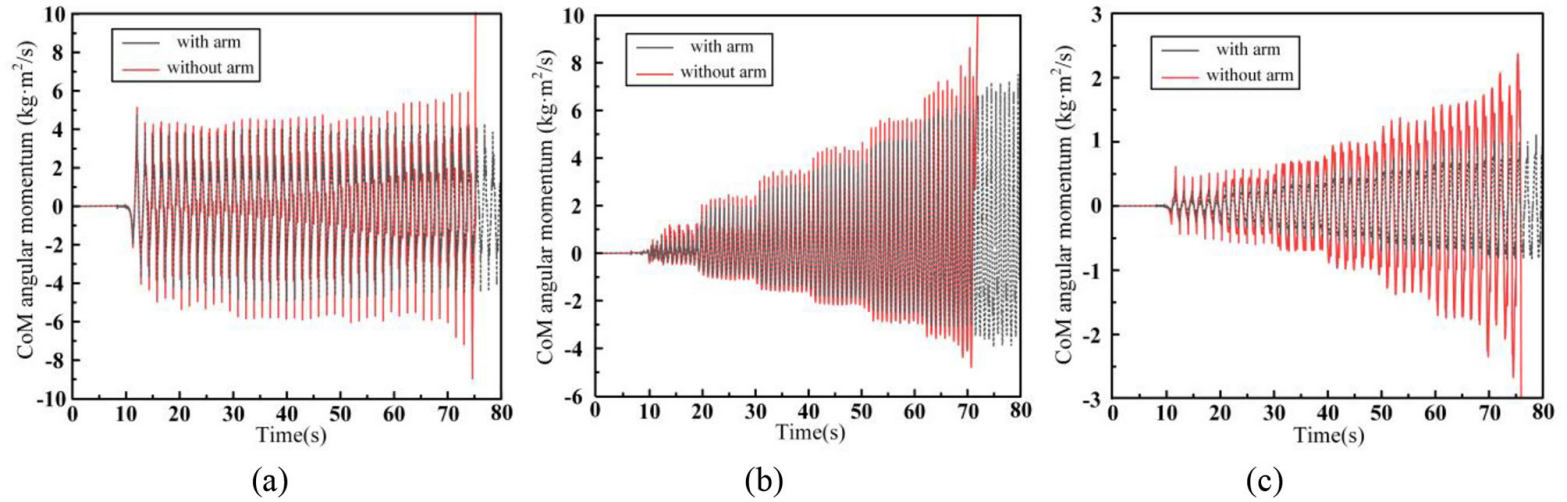
Humanoid robot centroidal angle momentum. **(A)** x-axis. **(B)** y-axis. **(C)** z-axis.

The walking control data for the humanoid robot with waist and arms swing is as follows:

In Figure 13A shows the tracking curve of the robot's desired velocity during walking, Figure 13B shows the swing trajectory of the key joints of the left leg, Figure 13C presents the motion trajectory of the robot's waist, and Figure 13D depicts the swing trajectory of the left arm joints during the walking process. After adopting the walking control framework proposed in this paper, the robot is able to gradually accelerate to 0.9 m/s with a regular increase of 0.15 m/s every 10 seconds and maintain stable walking at this speed. Compared to Scenario (1), where no waist and arm movements were employed, the maximum stable walking speed increased from 0.45 m/s to 0.9 m/s, proving the significant role of the waist and arms in improving the robot's walking ability. In contrast to Scenario (2), where no arm swing was used, the maximum stable walking speed increased from 0.75 m/s to 0.9 m/s, demonstrating that arm swing contributes to enhancing the robot's walking stability. From Figure 13B, comparing with Scenario (2) in Figure 11B, it is evident that as the walking speed increases, the range of leg movement also increases. When the arm swing is introduced, the robot's centroid momentum can be compensated by the arm swing, resulting in better overall stability. Therefore, the robot does not lose balance or fall due to changes in states during acceleration. In Figure 13C, the waist swing angle increases with walking speed. With the introduction of the arm swing, which enhances the robot's overall stability, the robot no longer needs to make large adjustments to the foot placement, making the waist movement more stable. Figure 13D shows that the swing amplitude of each arm joint increases with the walking speed, with the shoulder pitch angle and elbow joint exhibiting the largest movement range, similar to the natural arm swing trajectory in human walking. To highlight the influence of arm swing on the robot's state, the centroidal angle momentum trajectories for Scenario (2) and Scenario (3) are shown in Figure 14.

In the figure, Figure 14A represents the centroidal angle momentum along the x-axis, Figure 14B along the y-axis, and Figure 14C along the z-axis. For convenience, the directions of the centroid axes are aligned with the world coordinate system. The x-axis angular momentum is primarily generated by the motion of the robot in the y-z plane during walking, and it is mainly influenced by the foot lifting motion of the swing leg. The fluctuation range of this angular momentum is positively correlated with the height of the lifted foot. The y-axis angular momentum originates from the motion in the x-z plane, and it is primarily affected by the swinging motion of the leg. As walking speed increases, the required step length also increases, leading to a greater swing leg range and, consequently, an increase in y-axis angular momentum. The z-axis angular momentum arises from the motion in the x-y plane and is influenced by both the swinging leg and the waist motion. As walking speed increases, the range of waist and leg motion increases, resulting in an increase in z-axis angular momentum. A comparison of the centroidal angle momentum across the three axes shows that, compared to scenario (2), the introduction of arm swing coordination reduces the fluctuation range of the robot's centroidal angle momentum in all three axes. The most significant reduction is observed in the z-axis angular momentum. For instance, during walking at 1 m/s, the arm swing reduces the angular momentum deviation by 45.8%. In contrast, the x-axis and y-axis angular momentum deviations are reduced by only 8.43% and 9.32%, respectively.

This is primarily because our Upper-body WBC controller prioritizes CoM position control to avoid affecting the position of the Upper-body CoM during walking. This design ensures that the robot's arms remain near the symmetrical position of the torso in the body coordinate system. As a result, the robot's arms can only swing within a small range to compensate for angular momentum deviations along the x and y axes. However, the z-axis angular momentum can be more effectively compensated by the symmetrical forward and backward swing of the arms, which does not violate the upper-body centroid control task and provides sufficient range of motion.

It is important to note that the compensatory effect of arm swing on centroidal angle momentum is closely related to the robot's arm weight, moment of inertia, CoM position, and other dynamic parameters. The compensatory effect of arm swing on centroidal angle momentum varies for robots with different mass distributions.

## 5 Conclusion and future work

This paper proposes a dynamic walking control framework that combines an improved footstep planner and a whole-body coordination controller. In this framework, we first analyze the issues with traditional LIP-MPC and replace the conventional instantaneous velocity-based model with a LIP position-based analytical model to predict the robot's future walking states. This modification avoids the average speed tracking errors introduced by using instantaneous velocity as an iterative model. While maintaining the computational efficiency of MPC, it enhances the effectiveness of footstep planning in tracking the desired walking speed.

To improve the robot's dynamic walking capability, we introduce coordinated control of the robot's waist and arms during walking. The control strategy is inspired by human walking, where the waist increases the effective swing range of the foot, and the arms compensate for the centroidal angular momentum during walking. Based on this, we decouple the robot's upper and lower body, independently constructing dynamic models for each. We then design a whole-body coordination controller, which incorporates both a Lower-body WBC and an Upper-body WBC, to independently control the robot's lower and upper body. The hierarchical design of this controller resolves the challenge of multi-task coupling in multi-degree-of-freedom humanoid robots, which often leads to poor control performance with traditional controllers.

Finally, in our simulation experiments, we first conducted a comparative analysis between the proposed improved footstep planner and traditional methods in terms of desired speed tracking accuracy, proving the effectiveness of our method in improving speed tracking. Subsequently, we integrated all components into a complete control framework and validated it through the Dexbot robot simulation system. The results from three experimental conditions demonstrate that the introduction of waist and arm coordination significantly enhances the humanoid robot's dynamic tracking ability for the desired walking speed, proving the feasibility and effectiveness of the proposed framework.

In future work, we plan to incorporate a visual navigation system to control the robot's rapid traversal through complex environments and perform task-specific operations.

## Data Availability

The raw data supporting the conclusions of this article will be made available by the authors, without undue reservation.
